# Paving the Way: Contributions of Big Data to Apicomplexan and Kinetoplastid Research

**DOI:** 10.3389/fcimb.2022.900878

**Published:** 2022-06-06

**Authors:** Robyn S. Kent, Emma M. Briggs, Beatrice L. Colon, Catalina Alvarez, Sara Silva Pereira, Mariana De Niz

**Affiliations:** ^1^ Department of Microbiology and Molecular Genetics, University of Vermont, Burlington, VT, United States; ^2^ Institute for Immunology and Infection Research, School of Biological Sciences, University Edinburgh, Edinburgh, United Kingdom; ^3^ Wellcome Centre for Integrative Parasitology, Institute of Infection, Immunity and Inflammation, University of Glasgow, Glasgow, United Kingdom; ^4^ Wellcome Centre for Anti-Infectives Research, Division of Biological Chemistry and Drug Discovery, School of Life Sciences, University of Dundee, Dundee, United Kingdom; ^5^ de Duve Institute, Université Catholique de Louvain, Brussels, Belgium; ^6^ Instituto de Medicina Molecular João Lobo Antunes, Faculdade de Medicina da Universidade de Lisboa, Lisboa, Portugal; ^7^ Institut Pasteur, Paris, France

**Keywords:** genomics, transcriptomics, proteomics, functional screens, microscopy, apicomplexa, kinetoplastid

## Abstract

In the age of big data an important question is how to ensure we make the most out of the resources we generate. In this review, we discuss the major methods used in Apicomplexan and Kinetoplastid research to produce big datasets and advance our understanding of *Plasmodium, Toxoplasma, Cryptosporidium, Trypanosoma* and *Leishmania* biology. We debate the benefits and limitations of the current technologies, and propose future advancements that may be key to improving our use of these techniques. Finally, we consider the difficulties the field faces when trying to make the most of the abundance of data that has already been, and will continue to be, generated.

## Introduction

**Figure 1:**
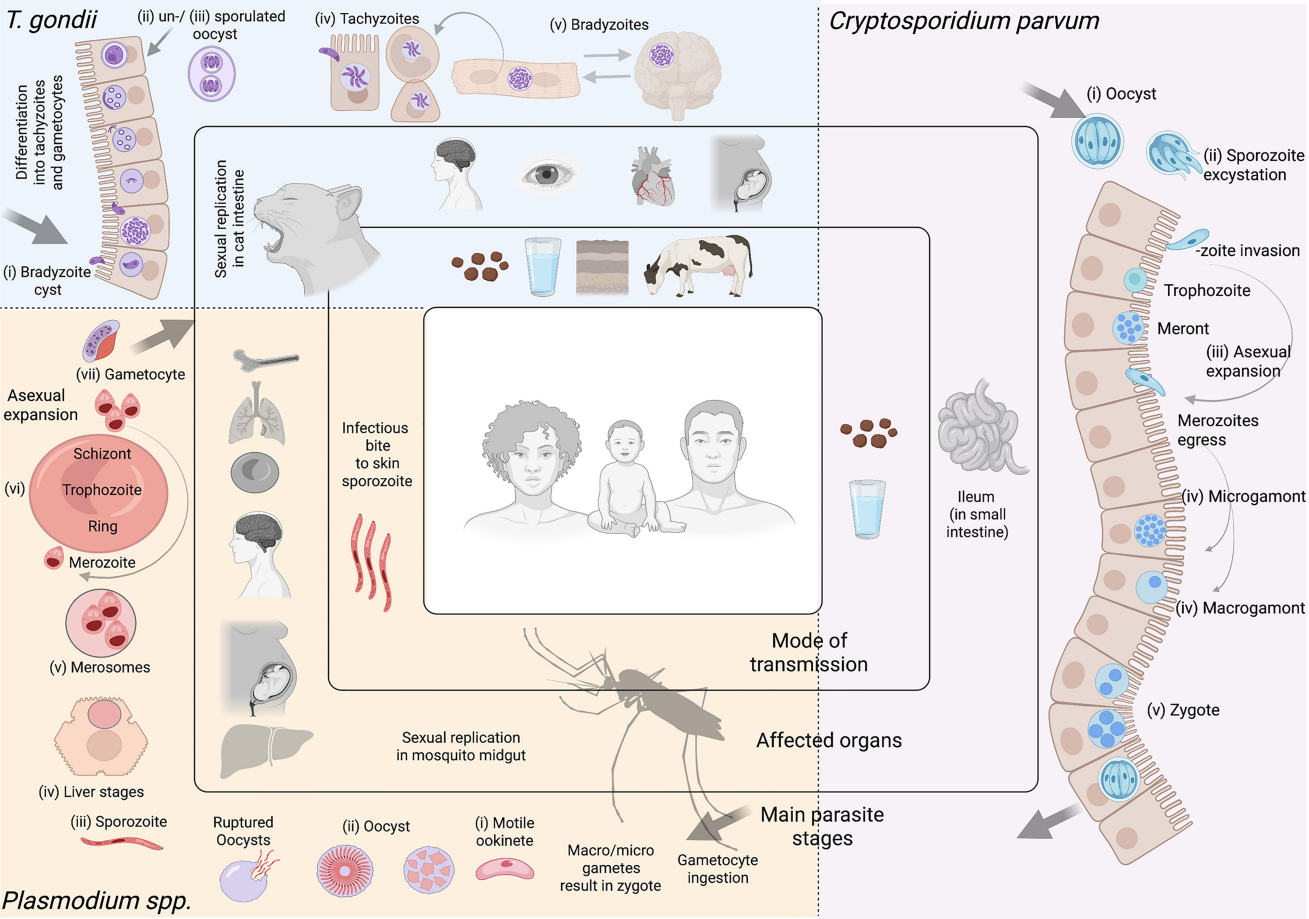
Apicomplexan parasites. Most apicomplexan parasites have complex life cycles with several developmental stages that occur in different hosts and in different organs or tissues within the host. While advances have been made to culture many stages of these organisms *in vitro*, some are restricted to short-term culture. For others, only a limited number of stages can be sustained. Equally, not all stages are amenable to genetic modification. In this figure we summarize main features of *Toxoplasma gondii, Cryptosporidium parvum*, and *Plasmodium* spp. ***Toxoplasma gondii.*** (i) After ingesting bradyzoite cysts from an intermediate host, the sexual developmental cycle of *T. gondii* occurs in the gut of felines culminating in the shedding of large numbers of (ii) un-sporulated oocysts in their faeces. Within a few days, oocysts sporulate in the environment and become infective. (iii) Intermediate hosts can become infected by consuming contaminated soil, water or plants. Once consumed, oocysts transform into (iv) tachyzoites in the host’s gut. Dissemination of a tachyzoite infection and repeated rounds of cell infection, replication and egress (lytic cycle) leads to a systemic infection. Immune pressure triggers some tachyzoites to form tissue cysts that contain (v) bradyzoites. In humans, tissue cysts most commonly found in skeletal muscle, the heart, the eyes and the brain. *T. gondii* tachyzoites are able to cross the placenta from mother to fetus. Reactivation of an encysted infection can occur upon immune suppression and ingestion of bradyzoite cysts by another intermediate host can transmit the infection (v to iii). Among these *T. gondii* stages, tachyzoites and bradyzoites can be cultured *in vitro* in large amounts. Tachyzoites are amenable to genetic modification. ***Cryptosporidium parvum.*** (i) Sporulated oocysts are excreted by infected hosts through faeces and transmission to humans usually occurs *via* contaminated water. Following ingestion, the parasite undergoes excystation, whereby (ii) sporozoites are released, and invade the epithelial cells of the ileum. Here, *C. parvum* undergo (iii) 3 cycles of asexual expansion, followed by (iv) sexual commitment to either micro- or macro-gametes. Fertilization occurs and results in the generation of a (v) zygote, which continues onto a sporulated oocyst. Some oocysts continue to reinfect the host while others are excreted. Cryptosporidium does not complete its lifecycle *in vitro* without the use of complex organoid systems. Even so, generating large amounts is limited. The sporozoite is the only stage used for transfections; to generate transgenic oocysts, transfected sporozoites must immediately infect an animal model or organoid. ***Plasmodium spp.*** Female *Anopheles* mosquitoes are responsible for transmitting *Plasmodium* spp. Mosquitoes are the definitive host, where *Plasmodium* undergoes sexual replication. This occurs in the mosquito’s midgut, where micro/macro-gametes generate zygotes which become motile (i) (ookinetes) and invade the midgut wall. Here they develop into (ii) oocysts. As oocysts mature, they rupture, releasing (iii) sporozoites which migrate to various locations in the mosquito, including its salivary glands. Following an infectious bite, sporozoites migrate from the dermis to the blood vasculature in humans. This allows them to reach the host liver, where they invade hepatocytes, and undergo a single round of (iv) asexual replication (by schizogony) resulting in the release of (v) merosomes filled with merozoites. Merosomes rupture in the blood circulation and release thousands of merozoites, which then infect red blood cells (iRBCs) and give rise to the erythrocytic stage of infection. Within RBCs, parasites develop into (vi) rings, trophozoites and schizonts. Mature schizonts rupture, releasing merozoites which invade other RBCs exponentially increasing the parasite mass. Some of these parasites will develop into sexual stages (called (vii) gametocytes). Mature asexual stages cause iRBC sequestration in organs including the brain, lungs, placenta, pancreas and adipose tissues, while sexual stages display preferential tropism to the bone marrow and other hematopoietic organs. Among these *Plasmodium* spp. stages, ookinetes, liver, asexual RBC, and sexual RBC stages can all be cultured *in vitro* in large amounts. Merosomes, rings, and schizonts are amenable to genetic modification. Note Large arrows in diagram refer to the completion of the cycle. Figure created with BioRender.com.

**Figure 2:**
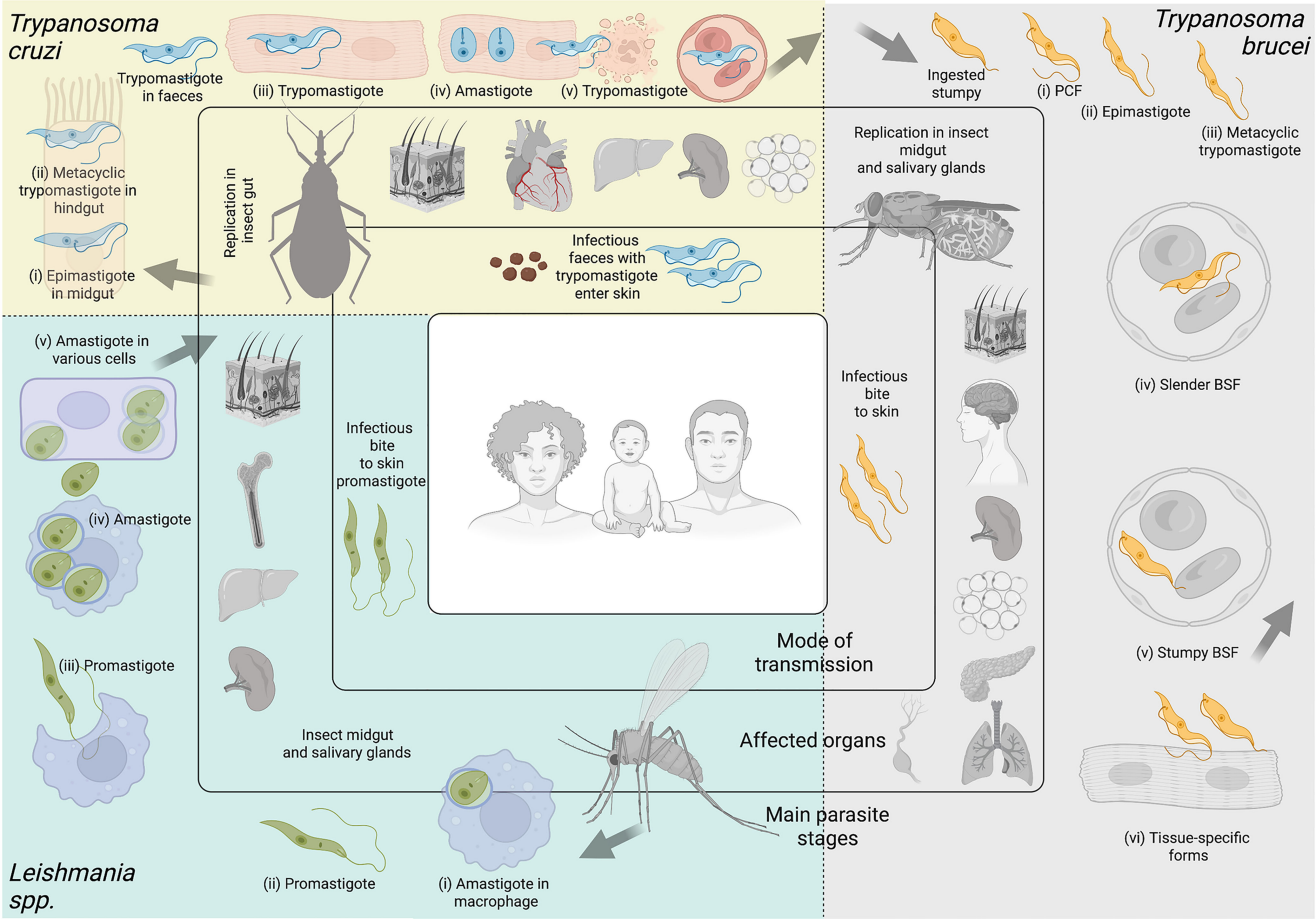
Kinetoplastid parasites. Kinetoplastid parasites have complex life cycles with various stages occurring in insect vector and mammalian hosts, and in different organs or tissues within their hosts. While advances have been made to culture several stages of these organisms *in vitro*, many are restricted to short-term culture. For others, only a limited number of stages can be sustained. Equally, not all stages are amenable to genetic modification. In this figure we summarize main features of *Trypanosoma cruzi, Trypanosoma brucei*, and *Leishmania* spp. ***Trypanosoma cruzi.*** Triatomine insect vectors of the genera *Triatoma, Rhodnius* and *Panstrongylus* become infected by feeding on infected blood (from humans or other animals). Ingested trypomastigote metacyclics transform into (i) epimastigotes in the insect’s midgut. These multiply and differentiate into (ii) metacyclic trypomastigotes in the hindgut. Infected vectors release trypomastigotes through their faeces on the host skin. Parasites enter the skin *via* wounds or mucosal membranes (such as through the eyes). Inside the host, (iii) trypomastigotes invade cells of a plethora of tissues, and transform into (iv) amastigotes which multiply and differentiate again into trypomastigotes, which are released from lysed cells. Some of these travel in the (v) bloodstream, and can be ingested by triatomine vectors upon a bite for blood feeding. The most commonly affected organ is the heart, but others, including the liver, spleen, and adipose tissues are invaded too, some of them becoming important parasite reservoirs. Among these *T. cruzi* stages, epimastigotes, trypomastigotes and amastigotes can be cultured *in vitro* in large amounts, and the whole life cycle can be modeled *in vitro*. Epimastigotes, trypomastigotes and amastigotes are amenable to genetic modification. ***Trypanosoma brucei.*** Tsetse flies (from the genus *Glossina*) become infected by feeding on infected blood (from humans and other animals). Within the fly’s midgut, *T. brucei* stumpy forms transform into (i) procyclic trypomastigotes (PCF). These multiply, egress from the midgut, and transform into (ii) epimastigotes, which can reach the fly’s salivary glands and continue to multiply. (iii) Metacyclic trypomastigotes are injected into the host skin during a bloodmeal. Inside the host, they transform into bloodstream form (BSF) trypomastigotes that can be (iv) slender or (v) stumpy forms, the latter of which rapidly transforms into procyclic forms in the tsetse midgut upon a blood meal. While slender BSFs multiply and thrive in the bloodstream, *T. brucei* is an extracellular parasite capable of invading multiple organs including the brain, spleen, adipose tissue, pancreas, lungs and lymphatic vasculature. These (iv) tissue-specific forms are relatively poorly understood. Among these *Trypanosoma brucei* stages, procyclics and BSFs can be cultured *in vitro* in large amounts, and the same stages are amenable to genetic modification. ***Leishmania spp.*** Phlebotomine sandflies become infected by ingesting infected cells during a bloodmeal. Within the sandfly, (i) amastigote forms of *Leishmania* spp. transform into (ii) promastigotes, which develop in the vector’s gut, and migrate to the proboscis. Infected sandflies transmit (iii) promastigotes during a bloodmeal. After entry into the skin, promastigotes are ingested by phagocytic cells (eg. macrophages and neutrophils). Within these cells, promastigotes transform into (iv) amastigotes, which multiply and (v) infect other cells. Depending on parasite and host factors, cutaneous or visceral leishmaniasis can result. For the former, the skin and soft tissues like the nose and mouth can be affected. For the latter, affected organs commonly include the spleen, liver and bone marrow. Among these *Leishmania* spp. stages, promastigotes, axenic amastigotes and intracellular amastigotes can be cultured *in vitro* in large amounts. Promastigoes and amastigotes are amenable to genetic modification. Note Large arrows refer to the completion of the cycle. Figure created with BioRender.com.

The global disease burden caused by Apicomplexan and Kinetoplastid infections is devastating world-wide. Among the Apicomplexans (**Figure 1**), *Plasmodium* spp. are responsible for a yearly estimate of 241 million malaria cases (WHO, 2021a); *Toxoplasma* gondii infects 30% of the human population (Milne et al., 2020); and *Cryptosporidium parvum* causes a yearly estimate of 44.8 million infections in children under 5 (Khalil et al., 2018). Among the Kinetoplastids (**Figure 2**), *Trypanosoma cruzi* affects 6-7 million people, mostly within the Latin American sub-continent where Chagas disease is most prevalent (WHO, 2021c); and *Leishmania* spp. cause an estimate of 700,000 to 1 million new leishmaniasis cases annually (WHO, 2021d). Additionally, *Trypanosoma brucei*, now responsible for less than a thousand yearly cases of Human African trypanosomiasis (WHO, 2021b) due to highly efficient vector control, and monitoring and surveillance programs, remains a current public health threat, as well as being a relevant model organism; These parasites continue to pose a major global threat, urging scientists to create and utilize novel molecular, cellular, pharmaceutical, bioinformatic, and imaging-based toolkits, to further our understanding of parasite biology, and develop new interventions to combat the diseases associated with these organisms.

Together, ‘omics’ technologies have shed light on vital aspects of parasite biology. Current high-throughput bulk ‘omics’ technologies have allowed us to characterise parasite genomes, transcriptomes, and proteomes at specific timepoints, to take a snapshot of the parasite population. Conversely, single cell technologies, including microscopy and single cell ‘omics’, allow us to probe variation within the population and describe asynchronous or rare events. Genomics and advances in genetic manipulation now allow high-throughput phenotypic screens to investigate gene function. Equally, while imaging has historically been a powerful tool for parasitology, increasing the throughput of microscopy methods holds great potential in the context of integrative ‘systems biology’. Another important aspect is the vast amount of data generated, and the capacity to analyse and effectively use this data. In parasitology, we have made significant strides in creating data sharing platforms (eg. VEuPathDB (Aurrecoechea et al., 2010; Amos et al., 2022). But as the complexity of these data increases, so do the challenges of data processing, integration, analysis, visualisation, interpretation and equitable sharing.

Here, we review seminal findings achieved in Apicomplexan and Kinetoplastid research by genomics, transcriptomics, proteomics, high-throughput functional screens, and imaging studies. We discuss the challenges, advances, and future directions of these technologies in the context of a key goal: how can we gain the most from the abundance of data that has already been, and will continue to be, generated?

## Genomics

**Figure 3:**
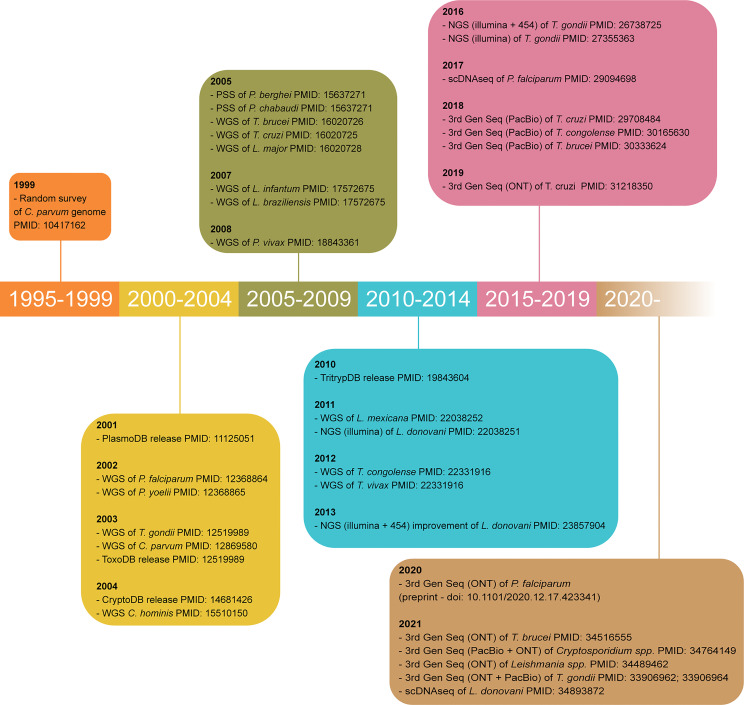
Timeline of major achievements in parasite genome sequencing. Only the oldest article using each technology for each parasite is cited. WGS, whole genome shotgun sequencing; PSS, partial shotgun sequencing; 3^rd^ Gen Seq, third generation sequencing or long-read sequencing; ONT, Oxford Nanopore technology; PacBio, Pacific Biosciences; PMID, PubMed identification number.

The early 2000’s were the advent of genome sequencing for Apicomplexan and Kinetoplastid research, as the genome sequences of *P. falciparum* (Gardner et al., 2002), *T. gondii* (Kissinger et al., 2003), *T. brucei* (Berriman et al., 2005), *T. cruzi* (El-Sayed et al., 2005), *L. major* (Ivens et al., 2005), *C. parvum* and *C. hominis* (Bankier et al., 2003; Abrahamsen et al., 2004; Puiu et al., 2004; Xu et al., 2004) were published. These laid the foundations for the high-throughput developments made since. Within less than two decades, the field has moved from Sanger and clone-by-clone sequencing to varied whole-genome shotgun sequencing technologies (**Figure 3**).

### Sanger Sequencing

For a long time, genome sequencing relied on the Sanger method (also called first-generation sequencing), which involves the random incorporation of chain-terminating fluorescently labelled dideoxynucleotide triphosphates (ddNTPs) during DNA replication and capillary electrophoresis to detect sequencing products (Gomes and Korf, 2018; Hu et al., 2021). Sanger sequencing is still widely used due to its cost-effectiveness ratio for gene-specific analysis or a small subset of genes, but it is impractical for analysing larger regions and has a low discovery power.

### Next Generation Sequencing

In the mid-1990s, sequencing by synthesis technology (SBS) was invented and provided the basis for next-generation sequencing (NGS) (also second-generation sequencing). The SBS approach relies on the incorporation of single fluorescently labelled dNTPs during DNA chain amplification. Illumina performs this in a parallel, high-throughput fashion, through cluster generation of DNA libraries by bridge amplification PCR (Hu et al., 2021). Together, all clusters in a flow cell could result in tens of millions of reads. Data generated by Illumina sequencing is highly accurate even for repetitive sequence regions and homopolymers. Compared to Sanger sequencing, NGS is high-throughput and provides higher sensitivity and coverage. However, because it generates short reads, it limits the analysis of structural variants, repetitive elements, and regions with a high GC content (Xiao and Zhou, 2020).

### Third Generation Sequencing

In the late 2000s, third-generation sequencing (3^rd^ Gen Seq, also known as long-read sequencing) was invented (**Figure 3**). The main 3^rd^ Gen Seq platforms are the Single-molecule real-time (SMRT) sequencing from Pacific Biosciences (PacBio) and the Oxford Nanopore technology (ONT). SMRT sequencing relies on the fixation of a single DNA polymerase to zero-mode waveguides (ZMW) with a single DNA template molecule. Through the ZMW, the SMRT cell can detect which single fluorescently-labelled DNA nucleotide is incorporated by the DNA polymerase and make the corresponding base call (Rhoads and Au, 2015; Hu et al., 2021). Instead of DNA polymerases, ONT uses the pore-forming protein α-hemolysin embedded in a membrane. This protein has the inner diameter of the size of a single strand of DNA. So, when current is applied to the membrane, the DNA strand moves through the nanopores, which alters the electric current and allows base-calling (Clarke et al., 2009; Brinkerhoff et al., 2021; Hu et al., 2021). 3^rd^ Gen Seq provides longer reads, allows detection of epigenetic markers, and can be portable, although error rates are still higher than NGS. Hybrid sequencing strategies have been implemented to improve sequence contiguity, error rates and affordability (Rhoads and Au, 2015).

### Apicomplexans

Comparative genomics studies in Apicomplexan parasites have been done particularly amongst *Plasmodium*, *Toxoplasma*, and *Cryptosporidium* genera (Carlton et al., 2002; Coulson et al., 2004; Carlton et al., 2008; Mazurie et al., 2013; Miotto et al., 2013). They have helped our understanding of population structure, evolutionary dynamics, epidemiology, and drug resistance mechanisms. Apicomplexan genomes are typically small (~8.5 to 130 Mb) (DeBarry and Kissinger, 2011; Blazejewski et al., 2015) and quite different from the typical eukaryotic genome. Their nuclear genomes are compact, shaped by substantial gene loss, have few transposable elements, and almost no synteny outside of their genus (DeBarry and Kissinger, 2011).

#### *Plasmodium* spp.

The genomes of *Plasmodium* spp. are haploid, both in cell culture and in the vertebrate host, with approximately 23 Mb in size and encode for ~5500 genes throughout 14 well-defined chromosomes (Pegoraro and Weedall, 2021). The biggest challenge for *Plasmodium* genome sequencing has been their extremely low GC content [21-23% compared to 56% in the mouse genome (Videvall, 2018)], although modern technologies have become less sensitive to this difference. The genomes of multiple species of human and non-human malaria parasites are readily available (Carlton et al., 2002; Hall et al., 2005; Carlton et al., 2008; Ansari et al., 2016; Auburn et al., 2016; Böhme et al., 2018). Interestingly, more than 60% of the genes predicted from the *P. falciparum* genome do not have homologs in non-*Plasmodium* organisms and they encode putative proteins of unknown function (Gardner et al., 2002; Neafsey et al., 2021). Their subtelomeric regions are rich in contingency gene families, many of which are large (>200 genes), hypervariable due to high recombination pressure, and involved in immune evasion (Barry et al., 2003) (the major variant surface antigens (e.g. *var*, *vir*, *pir* genes) (Singh et al., 2014; Ansari et al., 2016); the STEVOR genes, which are necessary for erythrocyte invasion of merozoites (Cheng et al., 1998; Niang et al., 2014); and the *rif* gene family, which are putative virulence factors (Fernandez et al., 1999; Araujo et al., 2018).

#### Toxoplasma gondii

*T. gondii* is the only species of the *Toxoplasma* genus (Tenter et al., 2000; Dubey, 2010). *T. gondii’s* genome is 65 Mb, encoding ~8,000 genes throughout 13 chromosomes [previously annotated chromosomes VIIb and VIII are now a single chromosome (Lorenzi et al., 2016; Berná et al., 2021)]. The *T. gondii* genome contains multiple repetitive and low-complex regions evenly distributed across chromosomes (Berná et al., 2021).

Classical genetic studies of the population structure of *T. gondii* revealed three clonal lineages (types I-III) in North America and Europe (Darde et al., 1988; Dardé et al., 1992; Sibley and Boothroyd, 1992). These share a common ancestor (Su et al., 2003), despite distinct pathogenicity in rodent models (Shwab et al., 2018). Genomics revealed a fourth clonal lineage mostly found in wild animals in North America (Khan et al., 2011). South American *T. gondii* strains display the highest genetic diversity of the species with recent genetic bottlenecks and lack of clonal structure (Sibley and Ajioka, 2008; Lorenzi et al., 2016). Genome-wide SNP analyses have shown recent genomic admixture among *T. gondii* clades, where large chromosomal haploblocks are inherited. Genomics has been crucial to elucidate mechanisms of transmission, host range and pathogenesis, particularly amongst *T. gondii* strains that have inherited conserved haplotype groups (Lorenzi et al., 2016). Genomics has also shed light on *T. gondii* virulence factors, such as the ROP proteins, which are major determinants of pathogenicity in mice (El Hajj et al., 2006). Some of these ROP genes have undergone local tandem duplication, locus expansion events and are under strong selection pressure by the host’s immune response (e.g mouse Immunity Related GTPases) (Peixoto et al., 2010; Steinfeldt et al., 2010).

#### *Cryptosporidium* spp.

There are currently 38 *Cryptosporidium* species reported that infect several host species (Feng et al., 2018). *Cryptosporidium* spp. genomes are ~9.1Mb in size, distributed across 8 chromosomes, and encoding ~4,000 proteins. Despite their much smaller size, *Cryptosporidium* spp. genomes have a gene density 1.8x higher than *Plasmodium spp*, a result of intron loss and reduction, intergenic regions shortening, and decrease of mean gene length (Keeling, 2004; DeBarry and Kissinger, 2011). Comparative genomics have shown that the most divergent regions of *C. parvum* and *C. hominis*, the most important human-infective species (Feng et al., 2018), are located near the telomeres. They are rich in transporters and surface-expressed genes, like other Apicomplexan and Kinetoplastid parasites (Bouzid et al., 2010; Widmer et al., 2012). These studies have also been key in identifying two new subtelomeric gene families that encode secreted glycoproteins [i.e. *C. parvum* specific proteins (Cops) and the *C. hominis* specific proteins (Chos)] (Bouzid et al., 2010), and are thought to play a role in the host-parasite interaction. Despite their name, advances in sequencing have shown that Cops is not species-specific, but rather conserved in *C. hominis* (Bouzid et al., 2013).

Most of the work done in this field has been based on SNPs found in the gp60 gene and revealed a very complex genetic structure (Tichkule et al., 2022). “Omics” analyses in *Cryptosporidium* have been delayed compared to remaining apicomplexans because the parasite is *quasi-*intracellular (i.e. intracellular but extra-cytoplasmic) throughout most of its life cycle; has a very small genome compared to the host cell, which reduces the power of direct sequencing; and long-term *in vitro* culture systems are technically challenging (Baptista et al., 2022). To date, the genomes of 15 species have been sequenced, 8 of which are annotated (Baptista et al., 2022).

### Kinetoplastids

#### *Trypanosoma* spp.

The genome sequencing of *T. brucei brucei* (Berriman et al., 2005) was followed by remaining *T. brucei* subspecies, *T. b. gambiense* (Jackson et al., 2010), *T. b. rhodesiense* (Sistrom et al., 2016), *T. b. evansi* (Carnes et al., 2015), and *T. b. equiperdum* (Hébert et al., 2017; Davaasuren et al., 2019). These genomes are ~32Mb in size and comparisons of these datasets have shown high synteny, large sequence homology and rare segmental duplications. However, these sequences, together with additional laboratory-adapted strains (Cross et al., 2014) and population isolates (Sistrom et al., 2014), have highlighted quite considerable diversity within the subtelomeres. The subtelomeres harbor multiple multi-copy gene families, of which the variant surface glycoproteins (VSG) are the most prominent. Comparative analyses of the genome sequences of *T. brucei*, *T. congolense* and *T. vivax*, have shown that each species has distinct mechanisms of generating antigenic diversity (Jackson et al., 2012; Silva Pereira et al., 2020) and thus have different strategies for establishing chronic infections. These genome sequencing projects have also allowed the determination of the cell surface phylome, a database of genes encoding cell-surface genes and their evolutionary relationships within the main African trypanosome species (Jackson et al., 2013). Moreover, whole genome sequencing of clinical isolates from Human Sleeping Sickness patients has shown that disease relapse results from ineffective parasite clearance by melarsoprol (Richardson et al., 2016). On a larger scale, studies of population genomics have shown the importance of sexual replication in African trypanosome evolution. It is now clear that, although certain African trypanosomes, like *T. b. gambiense* type 1 (Weir et al., 2016) and at least particular lineages of *T. vivax* (Duffy et al., 2009) evolve clonally, others such as *T. congolense* (Morrison et al., 2009; Tihon et al., 2017) and *T. b. brucei* (Peacock et al., 2011; Peacock et al., 2014), undergo hybridization. Likewise, genomic analyses of *T. cruzi* have highlighted how the rapid evolution of immune evasion-related gene families accounts for intraspecific variation (Wang et al., 2021). Population genomics and genetics have also been key to understand the population structure of Salivaria and Stercoraria trypanosomes (Franzén et al., 2011; Reis-Cunha et al., 2015; Jackson, 2016; Tihon et al., 2017; Callejas-Hernández et al., 2018; Silva Pereira and Jackson, 2018; Silva Pereira et al., 2018; Silva Pereira et al., 2020) and the identification of new trypanosome species and strains (e.g. *T. vivax*-like (Rodrigues et al., 2008; Rodrigues et al., 2017; Rodrigues et al., 2020), *T. suis* (Hutchinson and Gibson, 2015), *T. suis*-like (Rodrigues et al., 2020).

#### *Leishmania* spp.

Within the *Leishmania* field, research has focused on the *Leishmania* subgenus (i.e. *L. major*, *L. donovani*, *L. infantum*, *L. mexicana*). However, more recently, the subgenus *Viannia* has been attracting more attention, due to the growing recognition of the epidemiological importance of *Leishmania (V.) braziliensis*. With the exception of *L. amazonensis* (20Mb), *Leishmania* genomes contain 33Mb. Whilst genomics analyses of the *Leishmania* genus have revealed great chromosomal conservation (Ivens et al., 2005; Peacock et al., 2007; Rogers et al., 2011), studies of *L. braziliensis* and other *Viannia* species showed larger sequence diversity, differences in gene content, pseudogene number and chromosome copies, as well as novel mobile elements (Llanes et al., 2015; Valdivia et al., 2015; Ruy et al., 2019). The conservation of *Leishmania* genomes within different species contrasts with the extreme disparity in disease phenotype, tissue tropism, and clinical outcome. As in the trypanosome field, comparative genomics revealed a small number of highly-dynamic species-specific genes, as well as conserved gene families like the UDP-glycosyltransferases, that, despite their ancient origin, have diverged independently (Silva Pereira and Jackson, 2018). These examples of species-specific innovations are most frequent amongst the genes necessary for the coating and/or decoration of the parasite’s cell surface, and are likely to determine key pathways for parasite survival and adaptation in different hosts and environments. Recently, the field has used whole genome amplification of single cells and single-cell sequencing as means to detect aneuploidy mosaicism and reveal the specifics of its generation and evolution (Imamura et al., 2020; Negreira et al., 2022).

### Where Is the Genomics Field Going and What Remains to be Done?

A clear need in genome research is the improvement of reference genomes, both in terms of sequence contiguity and information. Long-read sequencing can help this because it resolves complex and repetitive regions and structural variants, and provides scaffolding evidence for already available genome sequences. Variations of these methods can also add information about epigenetic modifications, and genome architecture. It also facilitates sequencing of the minichromosomes and mitochondrial genomes. Furthermore, there is an urgent need for accurate and thorough annotation of reference genomes that support the increasingly sensitive transcriptomics and proteomics studies. Besides these points, genomics in the post-genomic era can answer key biological questions. Below we discuss two major examples.

#### Parasite Genomics Offer a Magnifying Glass Into the Evolution of Parasitism

The origin of Apicomplexans and Kinetoplastids is ancient, for instance, *Plasmodium*, *T. cruzi* and *T. brucei* diverged ~100 million years ago (Escalante et al., 1995; Stevens et al., 1998). Their genomes reflect that, by showcasing the expansions of contingency gene families and genome streamlining. This results from contractions in intergenic regions (Keeling, 2004; Panunzi and Agüero, 2014), loss of redundancy (Mendonça et al., 2011), and even some functional reduction (Bushell et al., 2017). *Cryptosporidium* spp. is an extreme example of genome compaction and reduction (Keeling, 2004), but this phenotype extends to remaining Apicomplexan and Kinetoplastid parasites, especially when compared to their free-living relatives. Genome sequencing of overlooked organisms can offer important insights into the development of pathogenicity and survival strategies, through the identification of parasite-specific innovations and/or loss of gene redundancy.

#### Comparative and Longitudinal Genomics Reveal the Microevolution of Parasite Lineages

Comparative genomics has been key to understanding the microevolution of parasite lineages, as a high-throughput method of population genetics. As the field progresses to single-cell genomics (Poran et al., 2017; Negreira et al., 2022) (**Figure 3**), long-read sequencing, and “post-genomic” tools (e.g. SNP barcoding panels (Daniels et al., 2008; Preston et al., 2014; Baniecki et al., 2015)), we will gain greater resolution into the dynamics of gene gain and loss, chromosomal reassortment, haplotype diversity and *de novo* mutations that may affect parasite fitness. Furthermore, these technologies allow a better understanding of parasite population history, geographical distribution, and the complex relationships between parasite and host co-evolution. They may also bring consensus to current debates in evolutionary biology, like the origin of *P. vivax* (Rougeron et al., 2020; Sharp et al., 2020). Finally, re-sequencing projects based on longitudinal sampling can offer a real-time overview of genome evolution dynamics, perhaps offering precious insights into how parasites respond to environmental pressures, including the everlasting pressure of host coevolution. Systematic, longitudinal field isolate sequencing can uncover complex genetic and evolutionary links that are not detectable at current resolution, whilst improving our understanding of genetic diversity, namely within contingency gene families.

### Challenges and Accessibility

A large bottleneck in the field of genomics has been the lack of analytical power to deconvolute complex data in a high-throughput manner. However, we have gathered a considerable set of computational tools that have streamlined the analysis of big data from parasite genomes. One of the most valuable platforms in parasitology is the VEuPathDB (https://veupathdb.org/), which integrates big data repositories across all ‘omics’ and multiple analytical and visualization tools. VEuPathDB has made an enormous impact on how parasitologists and vector biologists perform data mining. The specific impact and importance of VEuPathDB will be discussed in detail in the last section of this work. Nevertheless, there are other, more specialized tools include Companion, a web-based annotation tool (Steinbiss et al., 2016); VAPPER, a variant antigen profiler for trypanosomes based on diagnostic amino acid motifs and cluster of orthologs (Silva Pereira et al., 2019a), a *var* gene profiler based on DBL*a* domain sequence diversity (Barry et al., 2007), and CryptoGenotyper, which detects *Cryptosporidium* species from 18S/SSU rRNA sequences in mixed populations (Yanta et al., 2021). There have also been efforts to build biological sample repositories, where biological specimens or genomes from field isolates are archived, maintained, and made available to other researchers. Examples of these include the HAT Biobank (Franco et al., 2012), the TrypanoGEN biobank (Ilboudo et al., 2017), VAPPER (Silva Pereira et al., 2019a), and the Malaria Genomic Epidemiology Network (MalariaGEN) (Ahouidi et al., 2021). These are valuable sources of materials for future genome-wide projects. We take the view that future research will increasingly add to these tools, making genomic information readily available to all.

## Transcriptomics

Transcriptomics has rapidly expanded over the past four decades, with each new technology generating a wave of increasingly large data (Chambers et al., 2019), enabling the discovery of novel transcripts and splicing variants, UTR annotation, and the quantification of transcriptome-wide changes in gene expression in populations and, most recently, single cells.

### Technology/Methods

**Figure 4:**
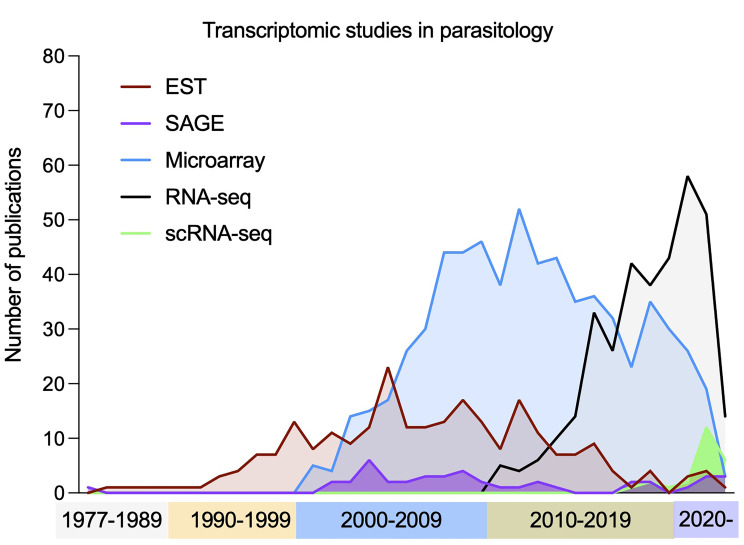
Publication of transcriptomic studies in parasitology. The use of transcriptomics has had a rapid increased over time, with early techniques (ESTs, SAGE and microarrays) becoming less frequently used in favour of bulk RNA-seq. Most recently the number of studies using scRNA-seq methods has increased to deconvolve mixed populations. Note: *Each term was searched for in publication titles and/or abstracts, along with at least one species of unicellular parasites included in the VEuPathDB database (Amos et al., 2022).

Key to the advancement of transcriptomics were complementary DNA (cDNA) libraries. Poly-adenylated mRNA is converted to cDNA *via* reverse transcription (RT) and cloned into bacterial plasmid vectors. Expressed sequence tag (EST) studies sequenced random library fragments and assembled them into partial transcriptomes (Sim et al., 1979), even without an available reference genome (Marra et al., 1998). The first use of the term “transcriptome” came when ambitions moved from identifying and sequencing transcripts to their quantification with SAGE (Serial Analysis of Gene Expression) (Velculescu et al., 1997). SAGE fragmented cDNA libraries and ligated short tags together before sequencing to improve throughput and qualification (Velculescu et al., 1995). The assembly of transcriptomes with these methods (and genomics) led to the use of microarrays, where a set of short oligomer probes are arrayed onto a solid surface and fluorescently labelled transcripts are hybridized. Microarrays require much lower input of mRNA compared to SAGE and can be used at higher throughput and lower cost, popularising their use in parasitology (**Figure 4**), but prior knowledge of the transcriptome is required.

Later, high-throughput RNA sequencing (RNA-seq) emerged (**Figure 4**). RNA is extracted and converted to a library of cDNA *via* RT and PCR amplification. During the process, adaptor sequences are ligated to facilitate sequencing with NGS (see *Genomics*). RNA-seq allows the boundaries of transcripts to be found at single-nucleotide resolution, has a higher throughput, higher upper detection limit, lower expense, lower requirements for starting RNA and more accurate quantification (Hrdlickova et al., 2017). Most recently, single cell transcriptomics (scRNA-seq) has come to the forefront. As RNA-seq requires RNA to be extracted from a population of cells, differences between individual cells are lost. scRNA-seq allows dissection of diverse and related cell types from a mixed pool. All approaches aim to add a unique cell barcode to transcripts from each cell during the RT steps (Hwang et al., 2018; Choi and Kim, 2019). The barcoded cDNA from multiple cells is then combined for the remainder of the library preparation. After sequencing, each read has the cellular barcode information allowing the transcripts to be grouped by cell of origin. Methods vary in how they isolate individual cells for the initial barcoding steps and are thoroughly reviewed elsewhere (Aldridge and Teichmann, 2020; Adil et al., 2021; Nayak and Hasija, 2021).

Transcriptomics is now frequently applied in parasitology (**Figure 4**), often for comparisons of perturbed and non-perturbed samples. In this section we focus on large studies of unperturbed parasites and offer perspectives of how transcriptomes can further benefit the field.

### Apicomplexans

#### *Plasmodium* spp.

EST and SAGE studies generated first transcriptomes of multiple *Plasmodium* spp. and their life cycle stages, uncovering novel genes and the prevalence of antisense transcription (Patankar et al., 2001). Microarrays and RNA-seq have since been used extensively to document the *Plasmodium* life cycle. Together these studies revealed the transcriptomic signatures of multiple aspects of the parasite’s biology, including the replicative stages, invasive stages and sexual stages. In particular, the developmental regulation of AP2 domain containing proteins has been uncovered, relating these key transcription factors to specific life cycle forms [reviewed in (Painter et al., 2011)].

**Figure 5:**
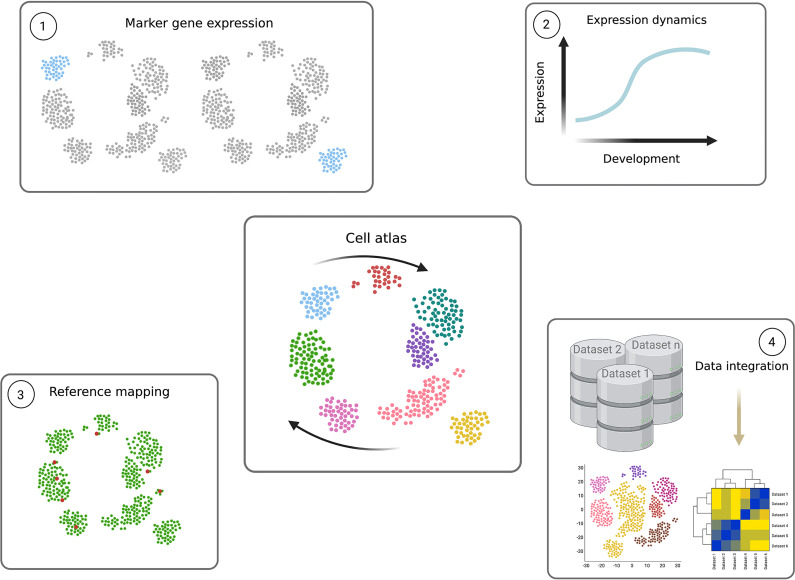
Power of single cell transcriptomics. Cell atlases (central figure) contain the transcriptomes of individual cells, organised according to transcript signature similarities and differences in low dimensional space. The result is a transcriptomic map descriptive of the system in question, which in parasitology can reflect the parasite’s complete life cycle (expressed as arrows). Individual parasite transcriptomes of the same life cycle form (single points) are positioned close together and, if captured, cells undergoing differentiation between different forms are positioned between the cell type clusters. These data can be mined and used as highly valuable resources in several ways. 1) Clustering analyses can be used to group similar cells in increasing resolution, often to identify life cycle forms. Differential expression analysis between clusters reveals novel marker genes, specifically expression in a particular cluster. 2) Pseudotime analysis can be performed to identify dynamic gene expression patterns across the life cycle. A path, or trajectory, is drawn through the cell atlas map connecting neighbouring cells and differential expression analysis is performed as a function of the trajectory. This reveals transcripts which change in level during the life cycle, and the exact expression pattern. Genes which peak in transitioning cells can reveal novel regulators. 3) The cell atlas can further be used as a reference to which query single cell transcriptomes can be mapped. For example, when only a few transcriptomes are available, or only those containing fewer transcripts per cell, mapping them to a high quality reference can identify their detailed position in the life cycle. 4) Transcriptomes of different genetically perturbed parasites, varied strains and even different species can also be mapped to the reference cell atlas through data integration methods. This allows detailed comparisons between datasets and across several cell types. Figure created with BioRender.com.

The “Malaria Cell Atlas” (Sanger, 2020) consists of individual parasite transcriptomes assembled into a map of the complete life cycles of *P. berghei* and *P. falciparum* and a partial atlas containing asexual blood stages of *P. knowlesi* (Poran et al., 2017; Reid et al., 2018; Howick et al., 2019; Real et al., 2021). scRNA-seq-generated cell atlases can be mined for dynamic gene expression patterns, to identify stage specific marker genes, and used as a high-quality reference onto which query transcriptomes can be mapped (**Figure 5**), as demonstrated in by mapping isolated *P. knowlesi, P. malariae, and P. falciparium* parasites to the *P. berghei* cell atlas (Howick et al., 2019). Beyond life cycle assembly, analyzing gene expression patterns using scRNA-seq has uncovered the transcriptional signature of the sexual committed schizont subpopulation (Poran et al., 2017; Brancucci et al., 2018; Ngara et al., 2018); insights into gametocyte formation without the need of schizont pre-commitment (Kent et al., 2018; Bancells et al., 2019); genes key for *P. falciparum* sporozoite infectivity to humans (Real et al., 2021); markers for *P. vivax* and *P. faliciparum* gametocytes; and *P. vivax* specific genes expressed in late schizont species mirroring the differences in RBC invasion between species (Sà et al., 2020).

Dual RNA-seq involves high depth sequencing of transcripts from infected host cells to analyse the host and parasite expression levels simultaneously. Although its application to *Plasmodium* research has enabled expression analysis of the host during infection, it remains difficult to assess parasite transcript changes due to the difference in host and parasite RNA levels in the sample (Lee et al., 2018). Dual scRNA-seq can now profile the transcriptomes of host cells and infecting parasites simultaneously, as performed with iRBC containing a single *P. falciparum* parasite (Poran et al., 2017). By identifying the subset of AP2-G expressing, sexually committed schizonts, the genes regulated by this master transcription factor could also be defined. Additionally, analysis of *var* genes expression challenged the previous dogma that *var* are mutually exclusive, as 3/17 individual cells expressed two *var* genes in parallel (Ngara et al., 2018).

#### Toxoplasma gondii

One of the most well studied *T. gondii* life cycle stages is the tachyzoite-to-bradyzoite differentiation step. As well as confirming the expression patterns of many genes identified with earlier technologies (such as bradyzoite specific secretory organelle proteins (Cleary et al., 2002)), the increased resolution of RNA-seq highlighted alternative splicing as a means of regulating expression, identified novel transcripts *via de novo* assembly and detected low expressed transcripts during this transition (Hassan et al., 2012; Chen et al., 2018; Garfoot et al., 2019). Oocyst maturation and subsequent reinfection of host cells by the sporozoites has been profiled using microarrays (Fritz et al., 2012) and SAGE (Radke et al., 2005), respectively. These provide the only profiles of transcript changes during these critical life cycle transitions to reveal stage-specific genes crucial for oocyst development and environmental survival. Dual RNA-seq of *T. gondii*-infected mouse forebrains uncovered differences between acute and chronic parasite metabolisms, with chronic stage parasites downregulating TCA cycle components but upregulating glycolysis (Pittman et al., 2014).

Around a third of detected *T. gondii* mRNA genes show upregulation in one of two “transcription waves”, peaking in the G1 phase or the S and M phases of the tachyzoite cell cycle (Behnke et al., 2010). The latter group of genes largely relates to apicomplexan-specific processes, mirroring the functional links between mitosis, generation of daughter parasites, and invasion organelles. scRNA-seq also revealed two distinct transcription waves, which were dissected into G1, S, mitosis and cytokinesis-associated genes (Waldman et al., 2020; Xue et al., 2020). The greater resolution revealed over 500 additional cell cycle-regulated genes, and those associated with phase-specific organelle development (Xue et al., 2020).

scRNA-seq has also highlighted unexpected heterogeneity during asexual tachyzoites-to-bradyzoites development (Waldman et al., 2020; Xue et al., 2020), including a subpopulation expressing a novel AP2 domain-containing gene and an intermediate transcriptome between tachyzoites and bradyzoites (Xue et al., 2020). SAG1-related sequence (SRS) proteins are expressed on the cell surface and are suggested to constitute an antigenic repertoire. Yet, only a small subset of parasites expressed *SRS* transcripts and did so with unexplained sporadic variation, the biological implications of which are yet to be uncovered (Xue et al., 2020). Notably, while these findings correspond to *in vitro*-derived cultures, comparative studies between culture-derived bradyzoites and bradyzoites isolated from mice has shown important differences (Pittman et al., 2014).

#### *Cryptosporidium* spp.

Efforts were first put into profiling the transcriptome with real-time-PCR targeting 3,302 *C. parvum* genes during *in vitro* infection of epithelial cells, revealing the differential expression of AP2 domain-containing genes in this Apicomplexan organism (Mauzy et al., 2012). RNA-seq has since been used to profile the *C. parvum* life cycle, revealing transcriptome signatures specific to the oocysts (specialized to survival and sporozoite delivery) and the asexual replicative intracellular stages (indicating high transcription and translation levels) (Lippuner et al., 2018; Matos et al., 2019; Tandel et al., 2019) and the sexual stages (highlighting genes involved in meiosis) (Tandel et al., 2019). Several AP2 domain-containing transcripts varied in expression, yet none were found to be exclusive to any one stage, suggesting redundancy (Lippuner et al., 2018). RNA-seq was also employed to improve the annotations of the *C. parvum* and *C. hominis* reference genomes (Isaza et al., 2015; Baptista et al., 2022). Analysis has yet to be performed of these data to compare the transcriptomes of the oocyct stages from each species, to the best of our knowledge. Exampling the use of older datasets, recently *C. parvum* ESTs (Wakaguri et al., 2009; Warrenfeltz and Kissinger, 2020) were mined to reveal extensive microRNAs (Ahsan et al., 2021) and RNA-seq to locate lncRNAs (Li et al., 2020).

### Kinetoplastids

Transcriptomics and genomics have revealed the unusual structure of Kinetoplastid genomes whereby one promoter precedes several genes that are transcribed as a polycistronic array and nearly all lack an intron-exon structure (Campbell et al., 2003; Haile and Papadopoulou, 2007). Transcripts are polyadenylated and a 5’ splice leader (SL) cap is trans-spliced. By specifically targeting the SL sequencing during library preparation, RNA-seq variations have been used to enrich *Leishmania* transcripts from host material (Haydock et al., 2015; Cuypers et al., 2017) and efficiently capture the 5’ ends of *T. brucei* transcripts (Kolev et al., 2015). This method has shown most genes have multiple SL and polyadenylation sites, and that these alternative sites can be used differentially between life cycle stages (Kolev et al., 2010; Nilsson et al., 2010; Siegel et al., 2010; Greif et al., 2013; Rastrojo et al., 2013; Jensen et al., 2014; Fiebig et al., 2015).

#### Trypanosoma spp.

Transcriptomics studies have revealed clear metabolism differences between life cycle forms of extracellular African trypanosomes. *T. b. brucei, T. vivax and T. b. gambiense* all show upregulation of glycolysis in BSFs in contrast to the tsetse stages which upregulate oxidative phosphorylation and the TCA cycle. Although *T. congolense* upregulates oxidative phosphorylation in procyclic and epimastigote stages, significant changes in glycolysis were not observed (Helm et al., 2009; Silvester et al., 2018). Analysis of tissue-specific *T. brucei* revealed further metabolic changes, as adipose resident forms further upregulate processes including glycolysis and purine salvage, and appear to uniquely express genes involved in fatty acid β-oxidation (Trindade et al., 2016). The intracellular parasite *T. cruzi* also exhibits strong metabolism switching between the mammal and triatomine vector (Minning et al., 2009). Interestingly, members of gene paralog clusters showed unexpected expression patterns during the life cycle, including amastins that were previously thought to be mainly exclusive to the amastigote stage appearing in insect stages (Minning et al., 2009).

During *Trypanosome* life cycles different cellular forms are often found in heterogeneous populations. scRNA-seq has been used to dissect mixed *T. b. brucei* populations and identify novel marker genes. These include slender and stumpy bloodstream forms generated *in vitro* (Briggs et al., 2021) and epimastigotes, gametes and metacyclics found in the tsetse fly salivary glands (Vigneron et al., 2020; Hutchinson et al., 2021; Howick et al., 2022). Additionally, midgut derived procyclic and proventricular forms have recently been profiled with scRNA-seq (Howick et al., 2022). If parasites transitioning between broad life cycle forms are also captured, trajectory analysis can be used to order individual parasites according to the gradual change in their transcriptome (**Figure 5**). Differential expression analysis is then used to find dynamic transcript changes during differentiation between life cycle forms. This approach uncovered genes peaking in expression during the slender to stumpy transition, including critical regulator ZC3H20 (Briggs et al., 2021), and highlighted upregulation of transcripts associated with translation and the ribosome during development of both stumpy (Briggs et al., 2021) and metacyclic forms (Howick et al., 2022). Interestingly, scRNA-seq profiling of parasites extracted from tsetse salivary glands highlighted that pre-metacyclics express up to 6 mVSG before selecting just one for monoallelic expression in mature metacyclics (Hutchinson et al., 2021; Howick et al., 2022).

RNA-seq (Archer et al., 2011) and scRNA-seq (Briggs et al., 2021) have profiled phasic expression during the cell cycle of *T. b. brucei* pinpointing the peak expression time of several genes including cdc2-related kinases and cyclins, pairs of which most likely control transition between cell cycle checkpoints.

#### Leishmania spp.

Transcriptomics has been applied to multiple *Leishmania* spp. to reveal gene expression signatures associated with specific life cycle stages. Gene ontology (GO) term analysis of these signatures from RNA-seq found several similarities between species, such as upregulation of cellular motility and ATP synthesis in promastigotes compared to amastigotes, and phosphorylation upregulation in mammalian infective metacyclic and amastigote forms (Cruz and Freitas-Castro, 2019). Despite these similarities, only 12-35% of the differentially expressed genes have orthologs between *L. major, L. mexicana* and *L. braziliensis* (Cruz and Freitas-Castro, 2019), indicating clear differences in the life cycles of these species yet to be fully explored. RNA-seq revealed further molecular differences between morphology-defined forms, including the subtypes of the promastigotes (Inbar et al., 2017; Coutinho-Abreu et al., 2020). The transition from procyclic through nectomonad to metacyclic *L. major* was associated with downregulation of the cell cycle, consistent with reduced histone transcripts during *L. infantum* differentiation from procyclic to metacyclic. scRNA-seq has also been used to find transcripts unique to procyclic and metacyclic promastigote *L. tropica* in culture, and revealed differences in metacyclic formation between different strains in log-phase growth (Louradour et al., 2022).

Dual RNA-seq has also revealed that *L. major* and *L. amazonesis* both alter transcriptomes very early in macrophage infection, with little change observed in either parasite or host once parasites are in the intracellular niche, and uncovering genes involved in survival (Fernandes et al., 2016). Comparison of *L. donovani* dual RNA-seq additionally revealed putatively key virulence genes, including adenylate cyclase which is known to inhibit innate immune response in *T. brucei* infection (Shadab et al., 2019).

### Perspectives and Future Directions

#### Completing the Life Cycles

Cell atlases of the *Plasmodium* life cycle are a highly valuable resource providing the transcriptomic signatures of each life cycle form as well as cells differentiating between forms. scRNA-seq was critical for gaining this level of resolution, as multiple transition steps occur asynchronously across the population and some life cycle forms are rare and only found as a sub-population which are difficult to isolate without marker genes. scRNAseq datasets (current and future) can provide a wealth of information including: identification of novel marker genes; dynamic gene expression patterns identifying transcripts peaking in specific cell types; and variation between cell types to identify developmental regulators (summarized in **Figure 5**). High quality cell atlases can also be used as a reference for other query single cell transcriptomes, for example, of a genetically altered parasite line, clinical samples or alternative species or strains. The lower number of cells needed, and the ability to analyse mixed populations also means many life cycle forms are now accessible for the first time. However, challenges still remain, namely, to obtain highly-viable cells and detect lowly-expressed transcripts. Hence, bulk RNA-seq is still a valuable tool because it provides greater depth when populations can be isolated, and remains significantly more affordable. To overcome these challenges, integrated analyses of scRNA-seq and bulk RNA-seq has been explored in other fields (eg. cancer and vascular biology), and is a possibility that remains to be explored in parasitology.

Here we have focused on parasite-derived data, yet transcriptomics can clearly be leveraged to understanding host-pathogen interactions in detail. As well as dual RNA-seq and scRNA-seq, spatial transcriptomics at near single cell resolution can now be used to prolife parasite and host cell transcriptomes within a tissue, and retain the spatial information (Rao et al., 2021). One such technology, Visium Spatial Gene Expression from 10x Genomics (https://www.10xgenomics.com/products/spatial-gene-expression), uses slides tiled with spots of adaptor oligos for RNA capture, where each spot has a specific barcode similar to scRNA-seq. When a tissue sample is laid over the slide it is imaged with microscopy and then the extracted RNA is barcoded according to its’ position within the tissue. Thus, each transcriptome can be spatially organised. Although the size of each barcoded spot (currently 55 µm) is larger than Kinetoplastids and Apicomplexans, this level of resolution will likely have a huge impact on our understand of parasitic life cycles within tissue niches and host responses.

Transcriptomics datasets could answer many key questions in the field, such as: how flexible is the African Trypanosome life cycle (Guegan and Figueiredo, 2021; Lisack et al., 2022; Matthews and Larcombe, 2022); how “persister-like” protozoa contribute to the life cycle and drug resistance (Barrett et al., 2019); and how intra- and extra-cellular parasites adapt to different microenvironments within their hosts (Silva Pereira et al., 2019b).

#### Improving Annotation

As discussed above, there is a clear need to invest in higher quality references with accurate annotations. This is important in transcriptomics as correct transcript (including UTR sequences) annotations are needed to generate accurate quantitative data. Transcriptomics can also aid genome annotation. Applying methods like SL primer RNA-seq in the Kinetoplastids to a greater variety of species, strains, and life cycle forms will allow researchers to select references much closer to the parasites investigated. Mining available RNA-seq data could also be highly valuable for defining missed transcripts, variable UTR boundaries and splicing variants not present in current references. The use of long-read transcriptomics/genomics can also begin to resolve multigene families which are prevalent among parasites.

#### Data Integration and Comparison

Integration of multiple datasets would be highly impactful. For example, several scRNA-seq studies have analyzed different stages of the *T. brucei* life cycle. Despite these all using a variety of methods, the raw data could be integrated as bioinformatic methods improve (Argelaguet et al., 2021) to provide at least a particle life cycle atlas, as demonstrated in the Malaria field. Population-based transcriptomics constitute a highly significant bank of data which, with upgraded analysis methods, could be combined, analysed and compared to gain significant insight into these pathogens’ biology. Here, we discuss only unperturbed parasite data, but these comparisons can clearly be extended to compare experimentally manipulated parasites. Lastly, “multi-omic” data integration would be hugely valuable to link transcript levels to protein levels and genomic features (Subramanian et al., 2020).

## Quantitative Proteomics

While mass spectrometry was being used in the 1990s for protein identification in parasitology, it was not until the early 2000s, once the respective genomes were published, that proteome datasets were derived. Quantitative proteomics remains an active field, as the advancement of mass analyzers has given rise to more sensitive mass spectrometers allowing for identification and quantification of low abundant ions in complex samples. These studies can generate large quantitative datasets where one can identify post-translational modifications, drug targets, life cycle differences, organellar compositions, among other applications.

**Figure 6:**
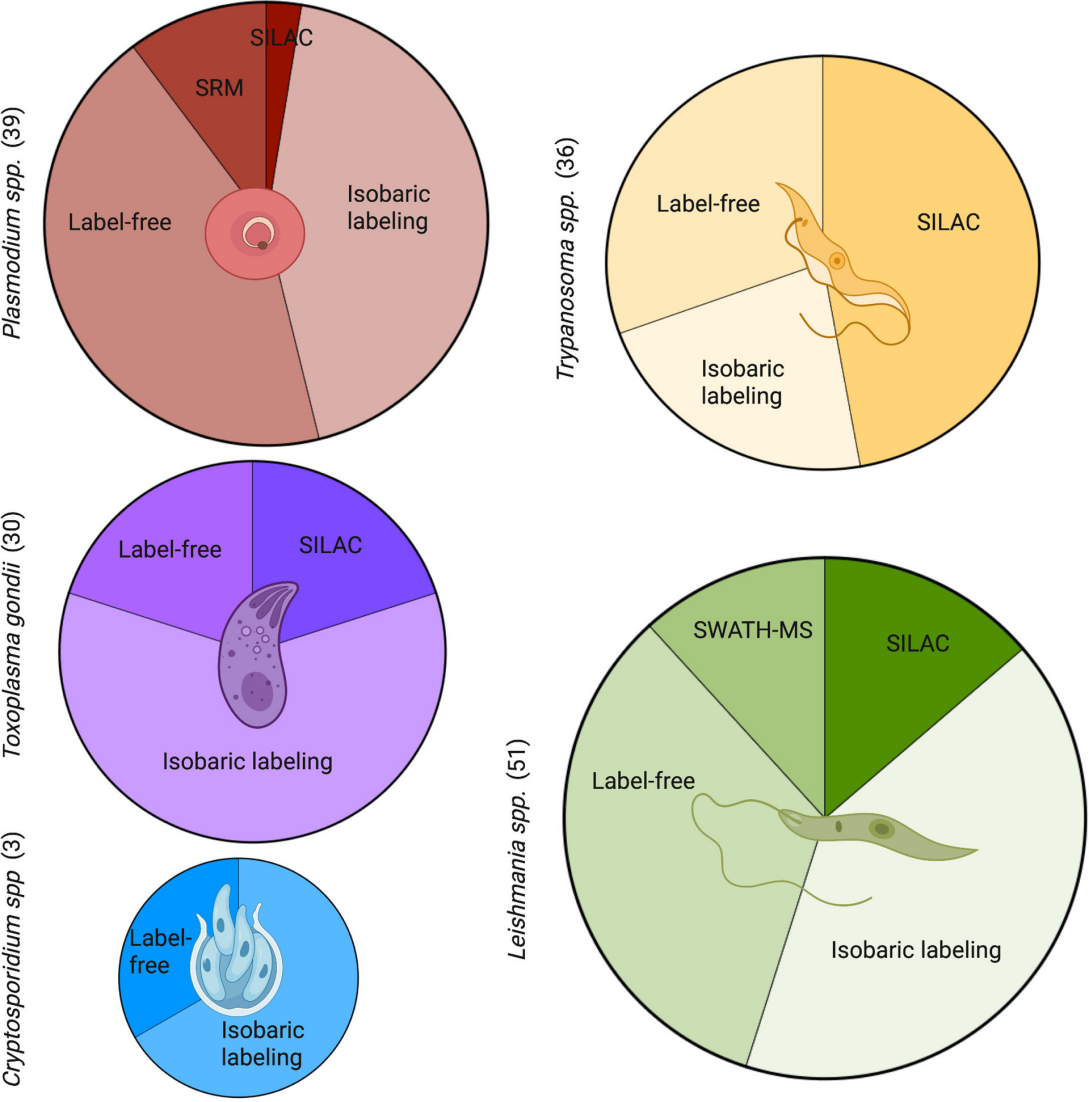
Commonly used quantitative methods to study proteomics in Apicomplexans and Kinetoplastids. A PubMed search was carried out for each genus with key terms for the proteomic methods. The number of publications by term and by parasite are shown in brackets. *Leishmania* had the most quantitative proteomic publications, followed closely by *Plasmodium*, *Trypanosoma*, and *Toxoplasma*. Both *Leishmania* and *Plasmodium* showed a larger diversity of methods with the inclusion of SWATH-MS and SRM, respectively. *Cryptosporidium* had the least amount of publications in quantitative proteomics and least diversity of methods. SRM, selected reaction monitoring; SILAC, stable isotope labelling by amino acids in cell culture; SWATH-MS, sequential window acquisition of all theoretical mass spectra. Figure created with BioRender.com.

Quantitative proteomics can be split into relative quantitation or absolute quantitation. In the Kinetoplastid and Apicomplexan fields, relative quantitation proteomics is more commonly used as seen by the number of publications (**Figure 6**). Here, we briefly describe some methods, consider their benefits and limitations, and discuss specific parasite-related examples and available datasets.

### Relative Quantitation

There are three commonly used methods in the Apicomplexan and Kinetoplastid fields to identify the relative abundance of proteins in a sample: Stable Isotope Labeling of Amino Acids in Cell Culture (SILAC), Tandem Mass Tag (TMT) or Isobaric Tags for Relative and Absolute Quantitation (iTRAQ), and Label-Free Quantitation (LFQ).

SILAC works by introducing a stable isotope variant of an amino acid, commonly lysine or arginine, that becomes incorporated during protein synthesis. Once cells take up the ‘heavy’ or ‘light’ isotopes, the cell lysates can be combined and proceed through to protein digestion, liquid chromatography and tandem mass spectrometry (LC-MS/MS). Some benefits of SILAC are the high and uniform labeling efficiency, minimizing sample loss by omitting peptide labeling, and labels are unaffected by protein purification steps (Ong et al., 2002). This technique is not ideal for life cycle stages where protein synthesis is inactive or bulk cell culture is difficult.

TMT and iTRAQ are two examples of isobaric labeling for mass spectrometry. They work similarly in that the labels used are of the same mass and are added after protein digestion to tag the peptides. Similar to SILAC, the individual samples are combined and run through LC-MS/MS, which allows for higher throughput in machine time and analysis with less run-to-run variability. TMT and iTRAQ currently have the ability to multiplex up to 18 and 8 samples, respectively. However, due to co-isolation and co-fragmentation there is a quantification distortion for low-abundant peptides. Additional statistical analysis or MS3 can be done to minimize this limitation.

LFQ differs from the previous techniques in that there is no label incorporation or tagging step. This can be beneficial as it allows for an unlimited number of samples to be compared, given that there is no limitation due to the number of available tags. However, this is at the expense of variation, technical variability, and throughput, as each sample is processed separately. Another benefit of LFQ, specifically compared with iTRAQ, is that the lower amount of protein loaded per run results in an average of 243 more identified proteins (with more than 1 peptide), with 34% increased sequence coverage (Patel et al., 2009). This is especially beneficial for organisms where it is difficult to acquire a large amount of material, as previously described.

### Apicomplexans

#### *Plasmodium* spp.

Although *Plasmodium* undergoes a complex life cycle in multiple hosts, most life stages are accessible enough to obtain sufficient material for proteomics, as seen with the hundreds of ‘protein expression’ datasets available on PlasmoDB (Aurrecoechea et al., 2009). There are 5300 predicted proteins in *P. falciparum* (Hall et al., 2005). Using SILAC, Nirmalan *et al*. (Nirmalan et al., 2004) effectively used isoleucine as their heavy isotype to quantify protein levels across the blood stages of *P. falciparum*. Isoleucine was the amino acid of choice because it was not made *de novo* from parasites or scavenged from the host, but efficiently taken up. Additionally, it is an abundant amino acid in *P. falciparum*, which allows for labeling to be present in most of the tryptic peptides (Nirmalan et al., 2004). Quantitative proteomics of the liver stages were done using *P. berghei* infected HepG2 cells. Over 100,000 merosomes were used per replicate with LFQ to identify 1188 proteins (with minimum 2 peptides) as the merosome proteome (Shears et al., 2019). Merosomes play a pivotal role as a ‘bridge’ between the liver and blood stages in the *Plasmodium* life cycle. Comparison with liver and blood *Plasmodium* proteomes showed both, significant similarities with both stages, and a subset of proteins unique to merosomes which warrants further investigation. In addition to merosomes, sporozoites at different maturation stages have been isolated from mosquitos to produce a surface proteome using LFQ in both *P. yoelli* and *P. falciparum* (Lindner et al., 2019). This allowed the identification of two distinct translational repression programs active during sporozoite maturation, that temporally regulate protein expression. This in turn governs major sporozoite life events in both, mosquito and mammalian hosts.

Additionally there are studies using host blood plasma samples to study host-pathogen interactions in patients with *P. falciparum* and *P. vivax.* Kumar et al. (2020) identified biomarkers for malaria severity using TMT labeling with LC-MS/MS. They found an up-regulation of cell-to cell adhesion-related host proteins in *P. falciparum* infections and not in *P. vivax*. This study generated a large dataset of infected host blood plasma data that has been deposited to the ProteomeXchange Consortium *via* the PRIDE partner repository.

#### Toxoplasma gondii

There have been over 20 proteomic studies with available datasets on ToxoDB (Kissinger et al., 2003). Some examples include using LFQ to develop a bradyzoite proteomic profile (Garfoot et al., 2019), SILAC to create the phosphoproteome (Treeck et al., 2014; Beraki et al., 2019), and using LC-ESI-HDMS (liquid chromatography, electrospray ionization, high definition mass spectrometry) for absolute quantification of the secretome of tachyzoites (Ramírez-Flores et al., 2019). To identify differences across tachyzoites, bradyzoite-containing cysts, and sporulated oocysts, Wang et al. used iTRAQ with LC-MS/MS and found 6285 proteins across the 3 stages, with hundreds being differentially expressed (Wang et al., 2017).

Most recently, a study using hyperLOPIT, a method that uses ultracentrifugation to separate subcellular structures prior to TMT labeling, created a comprehensive proteomic dataset of subcellular compartments in the extracellular tachyzoite. In this study, Barylyuk et al. (2020) were able to match 1916 proteins to known compartments within the tachyzoite. Less than 20% of the matched proteins had a clear, defined function, stressing the significance of this dataset in providing compartment composition for *T. gondii* as well as all Apicomplexans.

#### *Cryptosporidium* spp.

Since standard proteomic methods demand a highly concentrated protein sample, most of the stage-specific proteomes for *Cryptosporidium* are lacking. While there are not as many datasets available for *Cryptosporidium* as the other parasites, there are a few data sets available for the mammalian pathogen *C. parvum* on CryptoDB (Puiu et al., 2004) from the early 2000s identifying proteins in the intact oocyst, excysted oocyst and sporozoites (Truong and Ferrari, 2006; Snelling et al., 2007; Sanderson et al., 2008). These are currently the only life cycle stages where it has been feasible to collect enough material, as they are shed from large animal models, to perform proteomic analyses. Complementing the original proteomic data, there has been a quantitative study using iTRAQ with LC-MS/MS to compare sporozoites, intact oocysts, and excysted oocysts finding 302 proteins total (Snelling et al., 2007). Comparing the same *C. parvum* stages, Sanderson *et al*. (Sanderson et al., 2008) used 3 approaches (MudPIT, gel LC-MS/MS, and 2-DE) to maximize coverage. In doing so, they identified 1237 unique proteins that map to 32% of the predicted proteome. *C. parvum* IOWA II has 3894 protein coding genes (Puiu et al., 2004).

Recently, using the bovine parasite, *C. andersoni*, with TMT labeling, 1786 proteins were identified in the oocysts and sporozoites, of which 17 were differentially expressed between excysted and intact oocysts (Li et al., 2021a). *C. andersoni* oocysts are able to excyst solely with temperature change, unlike oocysts of *C. parvum* which require a combination of multiple stimuli (temperature, pH, cholates, proteases) (Smith et al., 2005), so comparisons of differentially expressed proteins between these species may be limited. Another recent study using label-free proteomics identified 231 proteins that correspond to intracellular stages of *C. parvum* at 36 hours post infection of HCT8s, an adenocarcinoma cell line (Li et al., 2021b). This study also identified 121 host proteins that were changed during infection. However, as *C. parvum* cannot complete its life cycle in HCT8s, there is a limitation in the conclusions we can draw from these host-pathogen expression differences.

### Kinetoplastids

#### *Trypanosoma* spp.

Reference genomes for *T. cruzi* and *T. b. brucei* show 9039 and 9660 protein coding genes, respectively (Jackson et al., 2012). A non-quantitative proteomic lifecycle of *T. cruzi* has been carried out and identified 2784 proteins, 30% of which overlapped across each life-cycle stage (Atwood et al., 2005). Early proteomic studies have had difficulty identifying all present proteins in samples and quantifying the identified proteins. However, later studies have used quantitative methods to quantify proteins in different life cycle stages. Using LFQ to study early metacyclogenesis identified 2720 proteins (with 2 unique peptides) in stationary phase epimastigotes and exponential phase epimastigotes (Avila et al., 2018). Ribosomal proteins were identified as some of the most upregulated proteins in the exponential phase, while metabolic enzymes were upregulated in the stationary phase (Avila et al., 2018). Also using LFQ, 114 proteins were identified to be differentially expressed in metacyclic trypomastigotes when compared to epimastigotes *in vitro* (de Godoy et al., 2012).

Various quantitative proteomic methods have also been used with *T. b. brucei.* TMT labeling of procyclic *T. b. brucei* identified 5325 proteins, of which 384 proteins were associated with cell cycle regulation (Crozier et al., 2018). Additionally using SILAC, Tinti *et al.* (Tinti et al., 2019) developed another interactive platform to compare protein turnover between blood stage forms and procyclic forms [platform access: https://tbrucei-ibaq-927.pages.dev/ and https://tbrucei-ibaq-427.pages.dev]. To study proteomic changes during the differentiation between slender and stumpy forms, stumpy forms were treated with citrate/cis-aconitate and samples were collected at 7 time points up to 48 hours post-treatment. LFQ analysis from these samples quantified 4270 ‘protein groups’, which were defined as groups of proteins that are indistinguishable by mass spectrometry from the identified peptides. Of these 1308 protein groups were found to be upregulated during differentiation and 157 protein groups were downregulated (Dejung et al., 2016).

#### *Leishmania* spp.

As both the amastigotes and promastigotes of *Leishmania* can be cultured *in vitro*, large amounts of material can be prepared for proteomic studies. Various datasets identifying proteins in the promastigote and amastigote forms have already been created (Brotherton et al., 2010; Nirujogi et al., 2014) and are available on tritrypdb.org (Aslett et al., 2010). Of the discussed protozoa, only in *Leishmania* was SWATH-MS used to identify differentially expressed proteins. Unlike the previously mentioned techniques, SWATH-MS is a data independent acquisition method. An example of this method is its use in identifying protein changes between 24 and 48 hours after *L. donovani* promastigote to amastigote differentiation. Routaray et al. identified 814 differentially expressed genes in the first 24 hours and 921 differentially expressed proteins at 48 hours post-differentiation (Routaray et al., 2022).

Another application of quantitative proteomics is thermal proteome profiling (TPP), which is an unbiased approach using TMT-labelling with mass spectrometry to identify drug targets. TPP studies generate quantitative datasets of bound proteins across a temperature gradient for all the soluble peptides in a sample. In this study Corpas-Lopez et al. used TPP to validate N-Myristoyltransferase (NMT) as a pharmacologically relevant target in *Leishmania* (Corpas-Lopez et al., 2019).

### Future Advancements

As single-cell sequencing and transcriptomics become more common, the interest in single-cell proteomics rises. However, even with the increased sensitivity of mass analyzers in recent years, the ability to accurately quantify peptide ions from a single cell remains difficult. Recent studies have tested a creative solution to circumvent this issue by adding a carrier proteome in addition to isobaric labelling (Cheung et al., 2021; Ye et al., 2022). However, carrier proteomes can bias which peptide ions are being identified and there is still a lower quality of MS data in terms of background signal (Ye et al., 2022). There is still a need for more sensitive instruments and greater multiplexing capacity in order to perfect performance of single cell quantitative proteomic studies. However, the development and utilization of single-cell proteomics within protozoans will be a way to reach the life-cycle stages that are not easily bulked up for standard MS, providing great insight into these yet understudied stages.

ONT has also been recently adapted for quantitative proteomics (Huang et al., 2019; Lucas et al., 2021). There are many benefits of using ONT for proteomics including lower cost, higher throughput potential, less maintenance, and higher portability compared to mass spectrometers. Together, these characteristics make proteomic studies more an accessible. Lower resolution is still a limitation, but one that is being addressed as the method and technology continue to be optimized. ONT for proteomics can be an accessible method for drug discovery in Apicomplexans and Kinetoplastids as well as identifying vaccine targets (Aebischer, 2014).

While both of these technologies are providing large advancements for the proteomics field, there is still work to be done in optimizing quantitative accuracy, resolution, and accessibility. These methods are being tested with large cells, such as HeLA or K562, as well as synthetic peptides (Cheung et al., 2021), but remain to be implemented in Apicomplexan and Kinetoplastid research.

## Functional Screening

**Figure 7:**
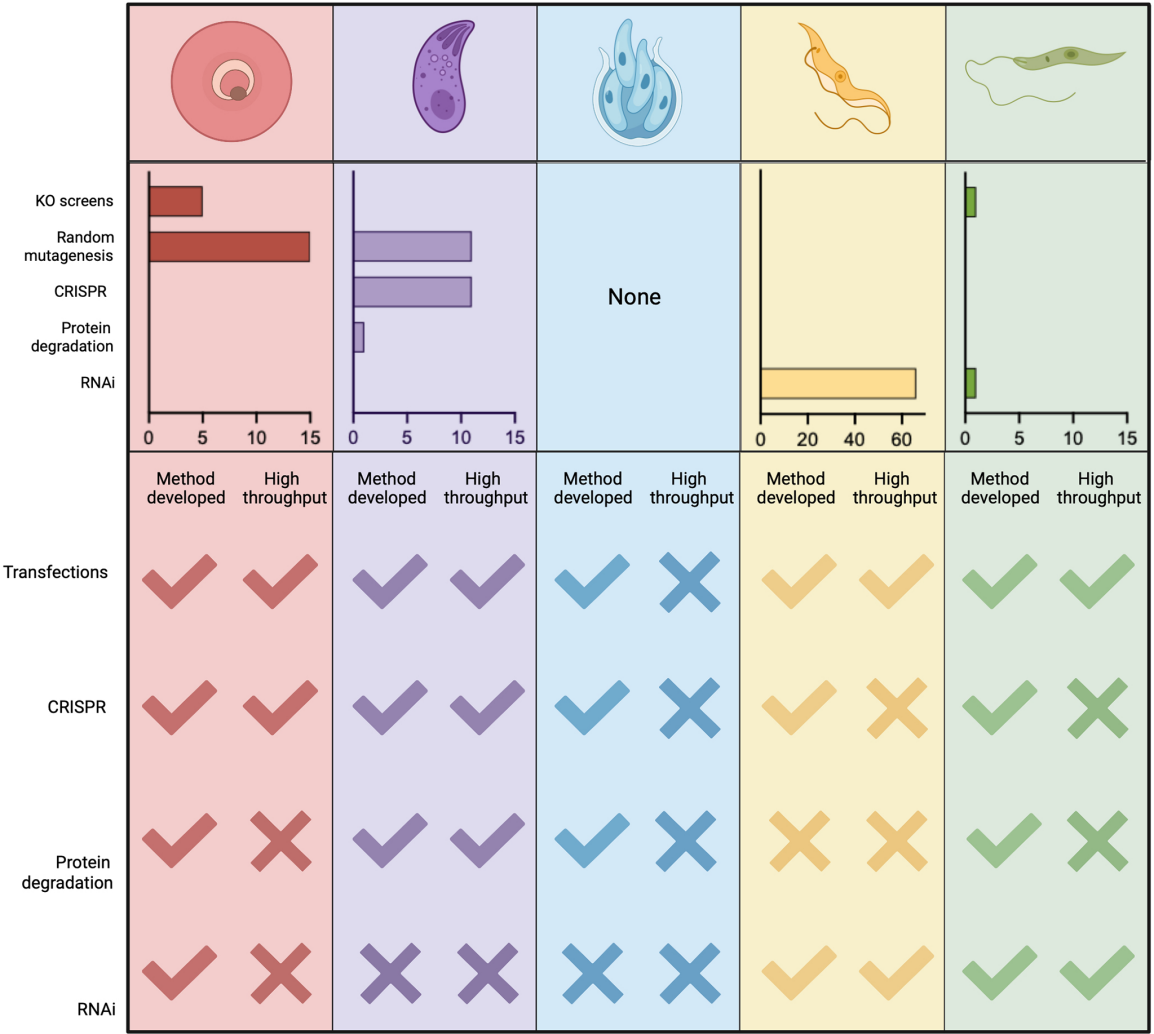
Publications using functional screens in Apicomplexans and Kinetoplastids. The number of functional screens completed in various Apicomplexans and Kinetoplastids is summarised. PubMed searches were carried out for each genus and screening method (y-axis) manual curation confirmed whether the method was used for screening rather than follow-up studies. In *Plasmodium* spp. random insertional mutagenesis (*pf)* and KO screens (*pb)* have been used extensively. In *T. gondii* chemical mutagenesis and, more recently, mutagenesis using CrispR libraries dominate. Functional screens in *Trypanasoma* spp. have been exclusively and extensively completed using RNAi. Few screens have been completed in *Leishmania* spp. and these are recent. Due to the lack of high throughput technologies in *Cryptosporidium* spp. no screens have been carried out on the parasites, only host screens. A summary of currently available technologies and their adaptation to high throughput, required for screening, is also shown. Figure created with BioRender.com.

Functional screens generally rely on the generation of mutant parasites *en masse* followed by specific screening assays to identify subpopulations that meet pre-defined criteria, before matching genotype to phenotype. The repertoire of tools for direct and conditional gene, mRNA and protein regulation developed and optimised for Apicomplexans (Briquet et al., 2021) and Kinetoplastids (Lander and Chiurillo, 2019; Horn, 2022) is extensive. Many of these technologies have also been scaled to enable functional screens (**Figure 7**).

### Genome Mutagenesis

Early screens used whole genome mutagenesis by chemicals, like N-ethyl-N-nitrosourea (ENU) and Ethyl methanesulfonate (EMS), or untargeted genome insertional mutagenesis, using transposons like PiggyBac, to generate mutants across the genome. Although key discoveries were made using these techniques (Radke et al., 2000; Morrissette and Sibley, 2002; Mordue et al., 2007; Farrell et al., 2014), which are a key resource, they are limited by difficulties in identifying and confirming specificity of mutations and the possibilities of multiple insertions. Signature tagged mutagenesis (STM) strategies have been used with some success to track mutants when combined with transposons (Mazurkiewicz et al., 2006) or chemical mutagenesis (Knoll et al., 2001).

There are several gene disruption approaches that target all/many genes within the genome (Gomes et al., 2015; Sidik et al., 2018; Baker et al., 2021; Horn, 2022). Additional methods have been developed to study the mutants within a population by: quantifying relative fitness of mutants (Gomes et al., 2015; Sidik et al., 2018), studying their localisation, and classification with high-content imaging (Li et al., 2022; Smith et al., 2022), and isolating subpopulations with specific phenotypes (Stanway et al., 2019; Harding et al., 2020). The generation of mass knockouts has been achieved using traditional recombination methods (Gomes et al., 2015; Bushell et al., 2017) and CRISPR Cas9-mediated mutagenesis (Long et al., 2016; Sidik et al., 2016; Baker et al., 2021).

**Figure 8:**
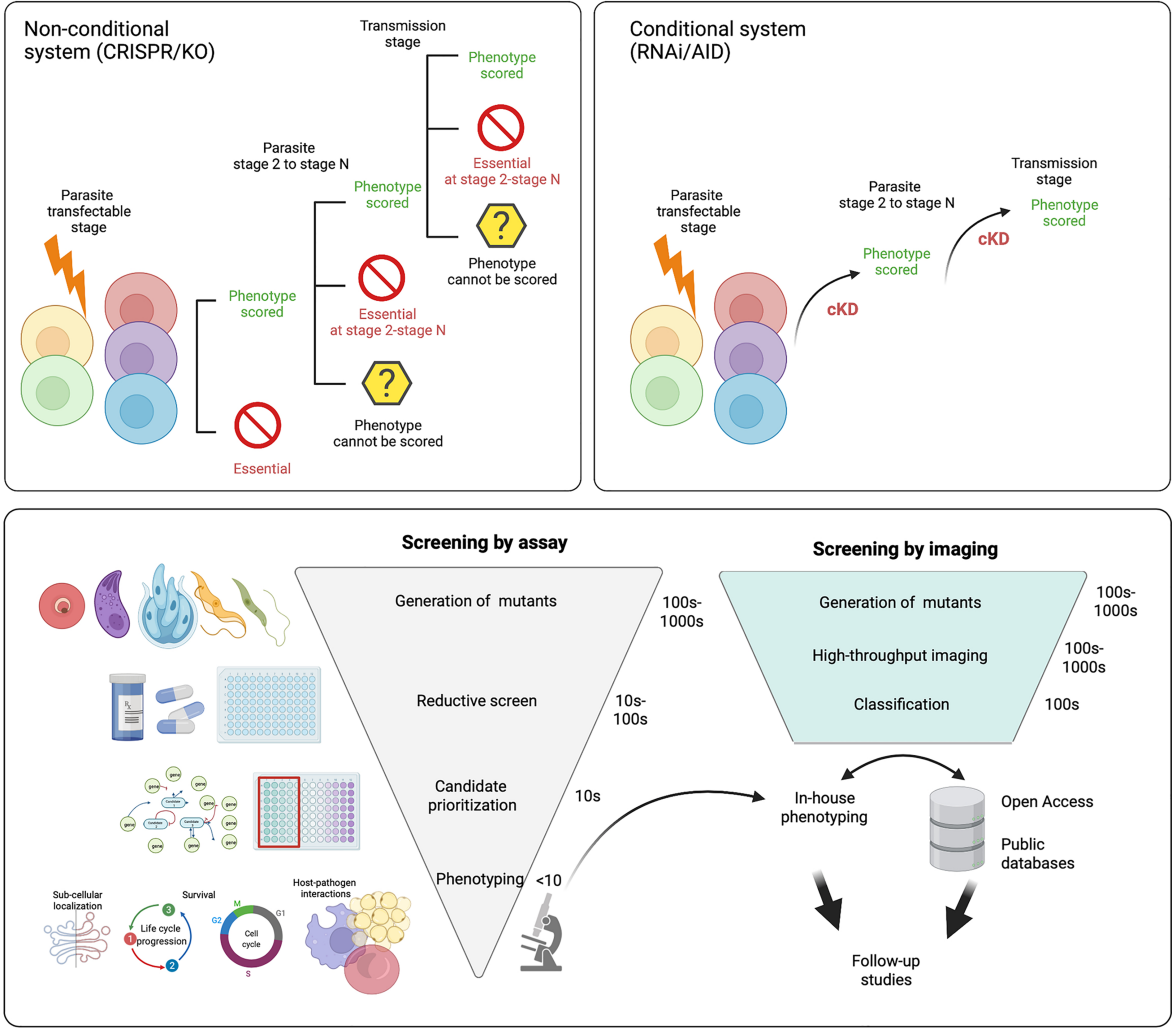
Future directions for functional screens. Most functional screens in Apicomplexa rely on non-conditional gene depletion methods to phenotype mutants. **Top left panel** After the gene(s) of interest has been disrupted only those that are dispensable for growth during the transfected stage can be phenotyped. With life cycle progression (through stages 2 - N) more mutants within the pool will be lost as they become critical for survival. This means even without reducing the population with selective pressure (eg. drug) the number of mutants within the pool that can be characterised is not complete across the life cycle. **Top right panel** In conditional regulation systems the means of downregulation are integrated (eg. the auxin tag for the AID system) following transfection. Of note it is likely a few candidates will not tolerate the tag and will be lost from the population. As downregulation can be induced across the life cycle, all mutants within the population can be characterised and none are lost due to prior stage essentiality. **Bottom panel** After generation of mutants, many functional screens rely on reductive assays to select mutants with a specific phenotype (eg. drug resistance). This is followed by further candidate prioritisation before in-house phenotyping of a small number of mutants, often re-derived as conditionally regulatable knockdowns to allow characterisation throughout the life cycle. If pools of mutants are instead characterised by high-throughput imaging, they can be classified based on tagged protein localisation or, mutant phenotype. Classification of mutants allows for in house phenotyping and open access data sharing distributes follow up studies throughout the field and improves equitability. Figure created with BioRender.com.

While many of these screens have been completed and have allowed us to parse out essential and non-essential genes in a variety of conditions, they have significant limitations, particularly in parasitic infections which have complex life cycles with only certain stages amenable to transfection and culture (**Figure 8**).

To combat this, several conditional systems have been developed; conditional gene excision or promoter inactivation can be achieved with conditional expression of site-specific recombinases [flp/FRT and Cre/loxP, (Combe et al., 2009; Andenmatten et al., 2013)] or by splitting the protein into nonfunctional subunits that regain functionality when fused together {DiCre, (Andenmatten et al., 2013) and splitCas [Li et al., 2022]}.

### RNA Regulation

As some kinetoplastids, like *T. brucei*, have functional, inducible RNA interference (RNAi) machinery, knockdown generation using short hairpin RNA (shRNA) is a widely used method for controlling expression. Other kinetoplastids with non-canonical RNAi mechanisms have been adapted for RNAi knockdowns (Horn, 2022) and even Apicomplexans, like *P. berghei*, can be adapted to express a minimal, non-canonical RNAi pathway (Hentzschel et al., 2020) enabling the use of RNAi to knockdown expression.

### Protein Regulation

Several methods have been used to regulate protein expression including the shield regulated destabilisation domain (DD), the trimethoprim regulated DHFR destabilisation domain (DDD) and the auxin inducible degron (AID) (Briquet et al., 2021). In these systems the protein of interest is often tagged and the degradation sequence added allowing localisation and confirmation of depletion. When combined with high-content imaging the localisation of tagged protein before and after induction of knockdowns can be used as a non-reducing screening method (see *Imaging* section). These degradation methods are limited to proteins accessible to the proteasome and those that can be tagged without interfering with function.

### Apicomplexans

#### *Plasmodium* spp.

The development of the PlasmoGem vector community resource provided a publicly accessible library to disrupt or endogenously tag genes across the *P. berghei* genome without having to generate vectors in house (Pfander et al., 2011; Gomes et al., 2015; Bushell et al., 2017).

The first screen completed using recombineering vectors determined the relative growth rate (RGR) for each gene knockout from pooled transfections. In this screen, 44.9% of all genes were defined as essential and 18% resulted in slow growth. Therefore, 63% of all genes were considered important for asexual growth *in vivo* (Bushell et al., 2017). Screening of function at subsequent stages of the life cycle is impossible once a gene confers a severe fitness defect (**Figure 8**). However, screening of non-essential genes, including slow-growers, has been completed using recombineering vectors during gametocyte development (Russell et al., 2021 (preprint)) and in the mosquito and liver stages (Stanway et al., 2019).

Although not yet adapted for high-throughput assays, several conditional systems have been developed in *P. berghei* and *P. yoelii* that may permit functional screening at all life cycle stages regardless of essentiality. The AID-system for protein degradation (Philip and Waters, 2015; Liu et al., 2021) could be integrated into the endogenous tagging pipeline of the recombineering platform (Pfander et al., 2011) to enable simultaneous localisation and control of protein levels by addition of IAA. While knockdown of AID tagged proteins has been achieved in the peritoneum of mice (Brown and Sibley, 2018), more biologically relevant knockdowns have yet to be demonstrated, possibly due to the high dose of IAA required to induce a knockdown and its toxicity for rodents in *in vivo* studies (Yesbolatova et al., 2020). The AID2 system uses ph-IAA to induce the knockdown and has been shown to function in mice with little toxicity (Saito and Kanemaki, 2021).

Although it was shown that the canonical RNAi machinery is not functional in *Plasmodium* spp. (Baum et al., 2009), recent work has supplemented the *P. berghei* genome with minimal RNAi machinery to allow control of expression at the mRNA level both constitutively and by using a stage specific promoter for temporal control of RNAi (Hentzschel et al., 2020).

Large scale functional genomic screens in *P. falciparum* have been hampered by difficulties with parasite genetic manipulation. However, transposon-mediated screens determined 50% of the genome is essential for *in vitro* growth of *P. falciparum* (Zhang et al., 2018), identified roles for post-translational modifications during blood stream growth (Balu et al., 2005; Balu et al., 2009; Balu et al., 2010), identified key responses to heat shock (Bronner et al., 2016), and uncovered novel insights into drug mechanisms of action (Pradhan et al., 2015). Although only tested in low throughput, CRISPR-Cas9 mediated genome editing has been adapted to *P. falciparum* and proof of integration has been successfully demonstrated for site-directed mutagenesis (Ghorbal et al., 2014; Nishi et al., 2021), epitope tagging (Kuang et al., 2017; Nishi et al., 2021), and gene replacements (Ghorbal et al., 2014). The major limitation for this as a high throughput technology comes from the length of homology arms required to ensure homologous repair (Aravind et al., 2003; Bryant et al., 2019).

As 50% of the *P. falciparum* genome has been identified as important for *in vitro* culture, conditional systems of regulation will be essential to elucidate the role of many genes during the blood stage and to uncover their roles at other life cycle stages. Several conditional technologies, including; DiCre, at the genome level; glmS and tetR, at the mRNA level; and DD-system, knocksideways (KS) (Birnbaum et al., 2017; Kudyba et al., 2021) and AID system (Kreidenweiss et al., 2013) at the protein level, have been shown to function in *P. falciparum* and may be able to expand the tools available for high-throughput functional analysis.

#### Toxoplasma gondii

Early genetic screens in *T. gondii* utilised chemical mutagenesis where pools of mutagenised parasites were cultured under different conditions to identify subpopulations that contained a specific phenotype, or the ability to survive in a set condition. While phenotype-specific screens have been informative, identifying protein mediators of egress (Black et al., 2000; Garrison et al., 2012; McCoy et al., 2017), signaling (Coleman and Gubbels, 2012), and structure (Morrissette et al., 2004), they are time-consuming requiring both sequencing and post-screen assays to identify the cause of the phenotype. Signature tagged mutagenesis (STM), which barcodes the population of mutants, has enabled more ready identification post phenotyping (Knoll et al., 2001; Mazurkiewicz et al., 2006).

The first CRISPR screen carried out in *T. gondii* targeted all predicted genes and quantified the *in vitro* fitness score for tachyzoites (Sidik et al., 2016). Since this primary genome-wide screen, subsequent screens have used the same library to identify genes important for other biological functions including growth in naive and interferon-γ (IFNγ) stimulated murine bone-marrow-derived macrophages (BMDMs) (Wang et al., 2020); resistance to dihydroartemisinin (Harding et al., 2020); and tolerance of oxidative stress (Chen et al., 2021). The library has also been adapted to target genes in a type II strain, which efficiently forms bradyzoites *in vitro* and *in vivo*. In this strain, fitness scores were calculated for *in vitro* growth and this was compared to the mutant’s ability to survive for 5 days *in vivo* (Young et al., 2019).

To enable the discovery of the role of genes that show a fitness deficiency or essentiality (at the tachyzoite stage) conditional systems have begun to be employed in functional screens, thereby allowing assays that probe function at other stages to be employed. A split Cas9 (sCas9) genome editing method, combined with a high-content imaging approach, was recently used to functionally group mutants based on actin dynamics and apicoplast segregation (Li et al., 2022). Another conditionally-regulated imaging-based screen used CRISPR-Cas9-mediated genome editing to introduce mNeonGreen and a minimal auxin-inducible degron (mAID) to an array of proteins (Smith et al., 2022. In these studies, using imaging to classify mutants led to the identification of several interesting phenotypes and genes of interest rather than continually reducing the mutants of interest to a suitable set of candidates as reductive assays have previously done.

#### *Cryptosporidium* spp.

Due to the lack of a robust *in vitro* model system, dependency on murine models for parasite passage, and lack of multiple selectable markers, *Cryptosporidium* is lagging behind the other apicomplexans and kinetoplastids in terms of high-throughput functional screens. However, there have been recent advancements such as the development of an accessible rodent model (Griffiths et al., 1998), air-liquid interface organoid culture system (Wilke et al., 2019), genetic tools (Vinayak et al., 2015) and validation of conditional gene regulation (Tandel et al., 2019) and protein degradation systems (Choudhary et al., 2020) that can advance the field. These could be used in conjunction with chemically mutagenised host cell lines to show different susceptibility to *Cryptosporidium* infection (Yu et al., 2017).

### Kinetoplastids

#### *Trypanosoma* spp.

The seminal RNAi screen in *T. brucei* procyclic stages was carried out in 2002 (Morris et al., 2002) to identify clones that were unable to bind the lectin concanavalin A (conA) including hexokinase 1. This study targeted the tsetse fly midgut stage, which was screened for *in vitro* fitness (Alsford et al., 2011), altered mitochondrial membrane potential (Verner et al., 2010) and tubercidin resistance mechanisms (Drew et al., 2003). This method paved the way for future screens improving on the laborious method for identification of mutants with the desired phenotype. To overcome slow mutant identification, RNA interference targeting sequencing (RITseq) is now used to quantify mutants within the population allowing fitness scoring (Alsford et al., 2011).

Improvements to the transfection protocols used on the cultured bloodstage form of *T. brucei* (Burkard et al., 2007) and the introduction of double stranded breaks (Glover and Horn, 2009) to further enhance efficiency has also enabled screens to be performed on this, more disease relevant, stage. To date, more than 60 screens have been performed on trypanosomes [reviewed in (Horn, 2022)] ranging from looking broadly at fitness (Alsford et al., 2011), to identifying mechanisms of drug resistance (Baker et al., 2011; Schumann Burkard et al., 2011) and biological processes like DNA repair (Burkard et al., 2007) and cell cycle progression (Schumann Burkard et al., 2013).

While high throughput screening for function using RNAi is established in multiple African *Trypanosoma* spp., *T. cruzi* lacks the machinery to use this system (DaRocha et al., 2004). Genetic tools for this organism are also lacking, with downregulation of genes being inefficient and plagued with issues of additional integrations due to gene amplifications (Burle-Caldas et al., 2015). While recent developments have generated *T. cruzi* reporter parasites that will enable imaging based screening (Costa et al., 2018) and have shown some success with CRISPR/Cas9 mediated gene editing targeting flagellar proteins (Lander et al., 2015), there have also been reports of Cas9 toxicity with continued expression (Peng et al., 2014). Although, transient Cas9 expression or targeting Cas9 itself alongside the gene(s) of interest could eliminate this issue (Peng et al., 2014; Lander et al., 2015). New techniques for the generation of repair constructs *en masse* (Xu et al., 2009) coupled with advances in axenic amastigote culture methods (Akutsu et al., 2019) could provide the opportunity to carry out high throughput screens in *T. cruzi*, an organism that has been so intractable to previous editing.

#### *Leishmania* spp.

Until 2015, double homologous recombination of laboriously generated vectors was the only way to screen for function in *Leishmania* parasites. However, supplementation of the genome with endogenous (*L. major, L. mexicana* (Beneke et al., 2017) and *L. braziliensis* (Espada et al., 2021)) or episomal (*L. mexicana*, *L. major* (Beneke et al., 2017), *L. tarentolae* (Turra et al., 2021), *L. donovani* (Martel et al., 2017) and *L. braziliensis* (Adaui et al., 2020) polymerase expression enabled the use of Cas9 mediated genome editing. Targeting a library of kinases in *L. mexicana* promastigotes, and using BarSeq to track mutants, showed that 79% of the kinome is dispensable for promastigote growth in culture, while 21% were refractory to gene knockout (Baker et al., 2021). Additionally, the requirement of these kinases was also evaluated *in vivo* and in the sandfly vector and the kinases were fluorescently tagged though this data has yet to be made publicly available (Baker et al., 2021). Recent developments to generate guide RNAs and homologous repair constructs in high throughput (Beneke and Gluenz, 2019), adapt barSeq technology (Beneke and Gluenz, 2020), and target both copies of a gene in a single transfection will all enable future high throughput screens in *Leishmania* spp.

In *L. tarentolae*, attempts have also been made to combine high-efficiency CRISPR-Cas9 mediated genome editing with the glmS conditional mRNA depletion system. However, only episomally expressed genes have been successfully knocked down (Turra et al., 2021). While this combination was unsuccessful, a conditional regulation system would prove valuable for studying the entire *Leishmania* spp. life cycle.

### What’s Next for Functional Screening?

Due to the complexity of parasite life cycles and the multistage requirement for many proteins, the use of conditional techniques to determine the function of genes and proteins seems imperative, and many conditional technologies are being developed for low- and high-throughput analysis (**Figure 7**). As some life stages cannot yet be cultured *in vitro*, characterisation of all life cycle stages requires screening systems to be functional *in vivo* (**Figure 8**). The development of AID2 makes this system an appropriate choice as previous work has shown efficient protein degradation *in vitro* and *in vivo* (Saito and Kanemaki, 2021). Until now, most functional screens have used reducing assays, characterised growth rate in specific conditions, or identifed a few candidates involved in a specific process or that confer resistance to a drug. These methods take an enormous number of mutants and filter them until the number of candidates is manageable for subsequent phenotyping analysis. The latter often relies on conditional regulation strategies to characterise function (**Figure 8**). Less reducing screening methods, such as coupling tagging and conditional mutant generation with high-content fluorescent imaging Li et al., 2022; Smith et al., 2022, or ultrastructure expansion microscopy (U-ExM) (Dos Santos Pacheco and Soldati-Favre, 2021) would allow testing a greater number of candidates and increase the descriptive detail of each, thus making the best use of screening outputs (see *Imaging* and **Figure 8**). Using unrestricted screens to classify mutants based on observations from high-content imaging would reduce the waste and/or duplication associated with reducing screening methods. Publication of these initial screens would also enable the sharing of preliminary phenotypes and allow a broader cohort of scientists, with different expertise, to follow-up on phenotypes of interest increasing equitable resource sharing.

When considering the classification of mutants following a functional screen, it is also worth noting that all techniques have limitations and interpretation of the data generated must be done cautiously. Descriptions of genes as essential must be treated with an awareness that this is only true for the life cycle stage being assayed and the growth conditions used. Furthermore, when transfecting pools of mutants, the loss of a mutant from the population may be more indicative of a severe growth defect and overgrowth, rather than an inability of the parasites to survive without this gene which may be uncovered with future phenotyping. Besides being a method of judicious gene selection, functional screens provide a wealth of data that should be utilised to its fullest.

## Imaging

**Figure 9:**
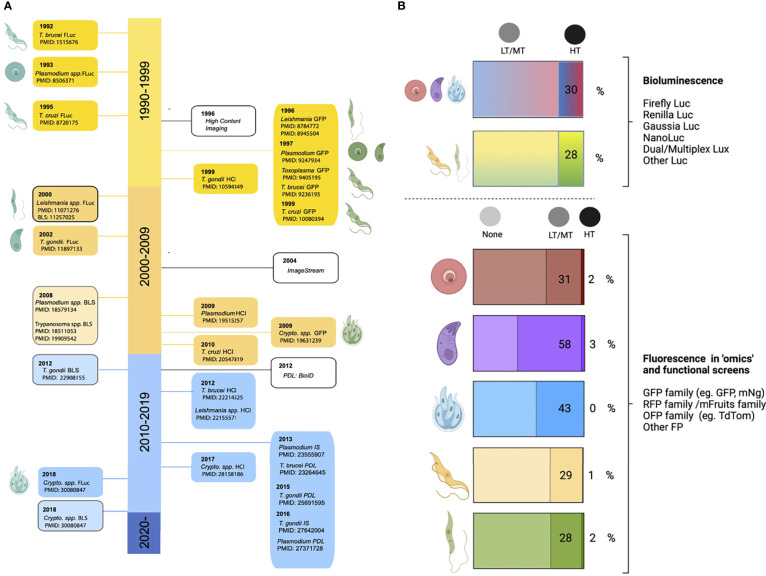
Microscopy usage in parasitology. **(A)** Timeline of key developments allowing high-throughput bioluminescent and fluorescent studies. The left side of the timeline shows the first transgenic bioluminescent lines created for each parasite (*Plasmodium* spp.*, Toxoplasma gondii, Cryptosporidium* spp.*, Trypanosoma* spp., and *Leishmania* spp.), as well as the first luminescence-based high-throughput screen (BLS) performed for each parasite. The right side shows the first generation of fluorescent reporter lines for each parasite, and the first use of high-throughput fluorescence imaging, including the use of high content imaging (HCI), ImageStream, and proximity-dependent labelling (PDL). **(B)** Top section shows studies using bioluminescent reporter parasite lines, specifying the percentage used in high-throughput screens (HTS) for Apicomplexans (*Plasmodium* spp.*, Toxoplasma gondii* and *Cryptosporidium* spp., Kinetoplastids (Leishmania spp., and Trypanosoma spp.). Bottom section shows the proportion of ‘omics’ and high-throughput screens using no imaging, low/medium throughput imaging (LT/MT), or high throughput imaging (HT). A PubMed search was performed for each genus, for all ‘omics’ methods, and each was explored to determine usage and throughput of microscopy. Figure created with BioRender.com.

**Figure 10:**
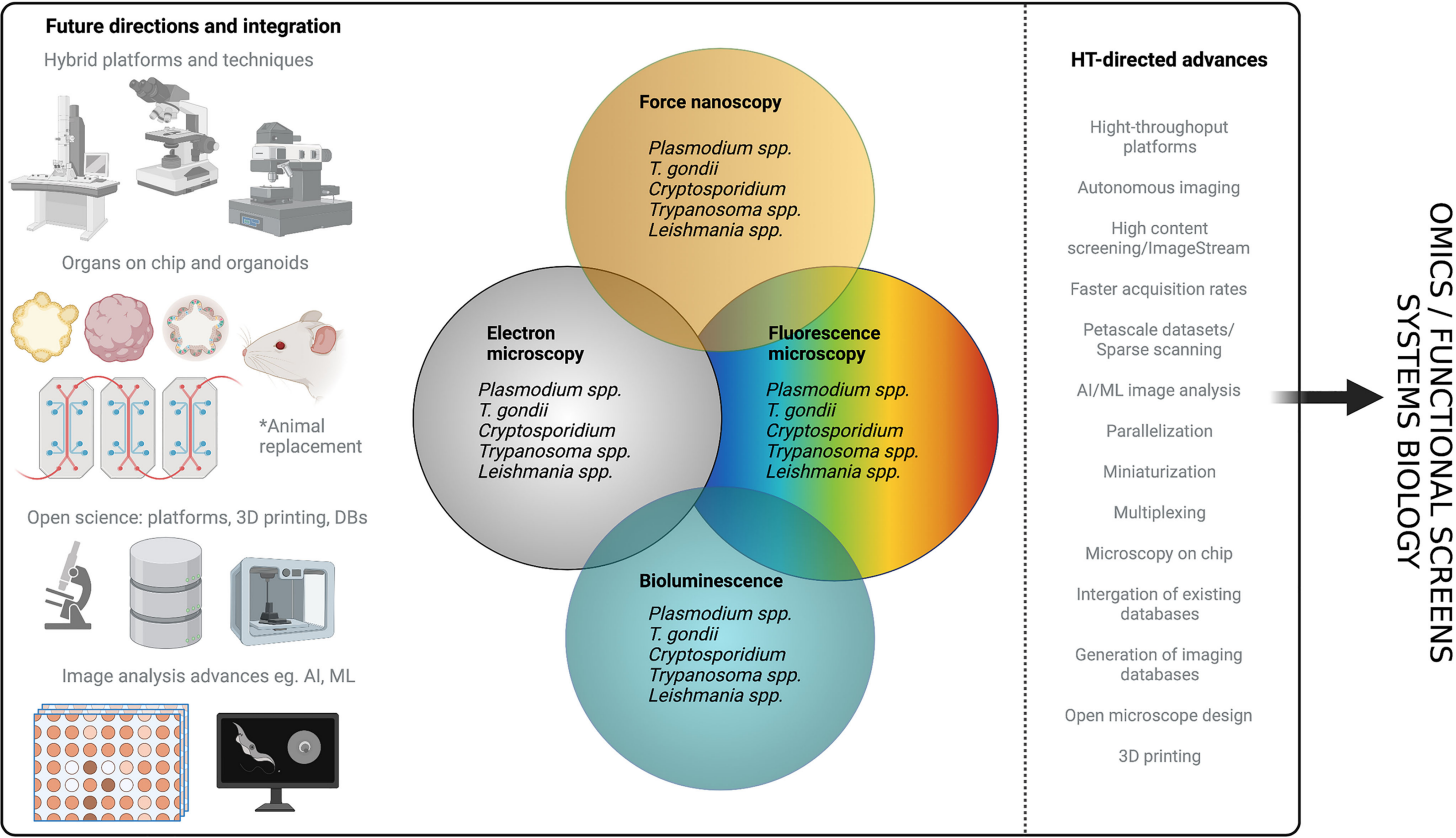
Microscopy current contributions and future directions. Multiple imaging modalities, including electron microscopy, fluorescence microscopy, bioluminescence, and force nanoscopy, have been extensively used in Apicomplexan and Kinetoplastid research. Efforts on technology development have led to the generation of hybrid imaging platforms (eg. combining electron and fluorescence microscopy in CLEM); the integration of cell culture, microfluidics, and bioengineering advances (eg. organoids and organs-on-chip, consistent with animal replacement and reduction) with imaging methods; the integration of 3D printing and robotics for the generation of versatile imaging platforms, including high-content imaging; and the integration of artificial intelligence for image analysis. Many of these improvements are consistent with a philosophy of open science, and have facilitated data-sharing and the creation of low-cost complex imaging equipment. Most of these have already been incorporated into parasitology research, but not in high-throughput modalities. Efforts towards increasing the throughput of conventionally low-throughput techniques are shown on the left column, and include autonomous imaging (whereby user input is not required, reducing human resource demands), miniaturization and parallelization, multiplexing, and faster acquisition methods. Equally, a bottleneck for microscopy-based research is image analysis. Incorporation of artificial intelligence and machine learning, and integration with open databases and open code are promising fields for parasitology. Together, these various elements will likely play a major role on the integration of imaging into ‘omics’ studies to understand parasite biology. Figure created with BioRender.com.

Microscopy has been pivotal for, and specifically for Apicomplexan and Kinetoplastid parasitology research (**Figure 9A**), including in the identification of most parasites. The microscopy toolkit in parasitology currently includes electron-microscopy, optical microscopy, force nanoscopy, and bioluminescence imaging among others (**Figure 10**). In this section, we will discuss i) the current role of imaging in the functional characterization of candidates arising from large screens using ‘omics’ technologies; ii) imaging methods currently used in parasitology as low-throughput and how they are being adapted to high-throughput; and iii) the advances in image analysis by the implementation of artificial intelligence.

### The Current Role of Imaging as a High Throughput Technology in Parasitology

The two main platforms used for high-throughput analysis in parasitology are bioluminescence imaging and high-content fluorescence imaging (**Figure 9**). Genetic modification of *Plasmodium spp., Toxoplasma spp., Cryptosporidium spp., Leishmania spp., T. cruzi*, and *T. brucei* to generate fluorescent/bioluminescent reporter lines (**Figure 9A**) has been key for high-throughput screens, both in the context of ‘omics’ and drug discovery (**Figure 9B**). Reporter lines have been vital to gain insight into phenomena such as parasite invasion, development, transmission, virulence and host-pathogen interactions, among others.

### High Content Imaging

High content imaging maximizes data capture, and can be adapted to multiple imaging systems, samples, cameras, and fluorophores. When coupled with robotics, high-content imaging allows monitoring of specimens over extended amounts of time, as well as imaging of a large number of samples with relatively little need for user input (Adams and Sjaastad, 2009). Although high-content imaging began as a method best-applicable to microplates, two relatively recent technologies, Opera Phenix™ - a high content screening device compatible with spheroid visualization and 3D printing, and ImageStream (George et al., 2004) have expanded this important toolkit. ImageStream combines the strengths of standard optical microscopy (at single cell level), and the sample sizes/statistical significance afforded by standard flow cytometry. Notably, high-content imaging can be used in parallel with assays such as RNA fluorescence *in situ* hybridization (RNA-FISH), and proximity labeling techniques [reviewed in (Kimmel et al., 2021)] such as BioID (Roux et al., 2012; Kim et al., 2016), APEX (Rhee et al., 2013; Lam et al., 2015) and TurboID (Branon et al., 2018). Adaptations in this respect include proximity ligation imaging cytometry (PLIC) (Avin et al., 2017) and Flow-FISH (Luiza-Batista et al., 2021). Proximity labeling techniques have been particularly useful for the study of the interactome and network mapping (protein-centric, RNA-, and DNA-centric interactomes) (Roux et al., 2018; Trinkle-Mulcahy, 2019).

### Bioluminescence Imaging

Bioluminescence imaging relies on the production of light by enzyme-catalysed reactions. In nature, bioluminescence is an evolutionary adaptation involving the natural production of light. Many bioluminescent substrates have been isolated, and the biochemical properties of their light defined (De Niz et al., 2016; Syed and Anderson, 2021). Multiple luciferase-expressing parasites have been generated (**Figure 9B**). Upon exogenous addition of relevant substrates (eg. luciferin, coelenterazine, furimazine), luminescence occurs, thus being detectable and measurable by ultra-sensitive cameras. Altogether, bioluminescence is a high-throughput method that has a simple output, facilitating quantitative analysis. While bioluminescence has been a vital tool for parasitology (Siciliano and Alano, 2015; Avci et al., 2017; Novobilský and Höglund, 2020), especially in the context of drug screening, important limitations of this technique include a) that it lacks the resolution to give insight into sub-cellular phenomena, which is often a main interest for candidate validation in ‘omics’ studies, and b) that the half-life of some luciferases does not allow for specific event captures. Nevertheless, a major advantage of bioluminescence beyond the context of high-throughput screening, is its applicability to *in vivo* screening (eg. rodent models) in a non-invasive manner, allowing for longitudinal studies to be performed. This, however, is beyond the scope of this review.

### Apicomplexans

#### *Plasmodium* spp.

Microscopy has allowed the investigation of *Plasmodium* invasion, development, deformability, pathogenesis, and genetic- and drug-screening (Gomes et al., 2015; Schwach et al., 2015; Brancucci et al., 2018; Duez et al., 2018; Kent et al., 2018; Tong et al., 2018; Stanway et al., 2019; Chia et al., 2021). A recent example of an ‘omics’ study directly incorporating high-throughput imaging to analyse *Plasmodium* liver stage development (Stanway et al., 2019) was based on a resource of individually barcoded gene knock out vectors (Pfander et al., 2011; Gomes et al., 2015; Bushell et al., 2017).

ImageStream has been used in high-throughput studies investigating anti-malarial drug effects against the *Plasmodium* digestive vacuole (Lee et al., 2014; Lee et al., 2016; Chia et al., 2017; Haridas et al., 2017), as well as to investigate the transcriptional signature and changes in parasite metabolism of *Plasmodium falciparum* sexually committed parasites (Brancucci et al., 2018). Furthermore, ImageStream and flow-FISH have been coupled to study gene expression in blood and liver stages of human malaria species (Luiza-Batista et al., 2021). Biotin-based proximity-labeling techniques have been successfully implemented to study various stages of *Plasmodium* (Kehrer et al., 2016; Khosh-Naucke et al., 2018; Schnider et al., 2018; Kimmel et al., 2021; Wichers et al., 2021) to investigate protein networks. The success of this implementation, and the potential for its coupling with large screen imaging methods (eg. ImageStream) holds great promise for the study of protein interactomes. Beyond fluorescence, several *Plasmodium* species have been engineered to express bioluminescent reporters across one or more of the parasite’s developmental life cycle stages. Main applications include the study of liver and blood stage development; *Plasmodium* strain-specific differences; stage-specificity of promoters and other regulatory elements; gametocyte development; functional studies of parasite proteins; parasite attenuation by genetic manipulation; anti-malarial activity of drugs (including extensive investigation of the Medicines for Malaria Venture (MMV) Malaria Box) (Khan et al., 2012; Cevenini et al., 2014; Vos et al., 2015; Ullah et al., 2017; Malebo et al., 2020); and investigating vaccine efficacy (Othman et al., 2017; Moita et al., 2021).

#### Toxoplasma gondii

Multiple *Toxoplasma gondii* fluorescent reporter lines have been invaluable for the phenotypic characterization of genetically modified parasites in terms of invasion capacity, growth, motility, survival/fitness, and host-parasite interactions (Bichet et al., 2016; Harding et al., 2016; Jacot et al., 2016; Sidik et al., 2016; Frénal et al., 2017; Guérin et al., 2017; Periz et al., 2017; Brown et al., 2018; Lentini et al., 2021). Examples of studies that have incorporated high-content imaging include the development of CRISPR-mediated tagging to study the *T. gondii* kinome (Smith et al., 2022). Another genome-wide phenotypic screen using splitCas9 in combination with high-content imaging, used indicator parasites to visualize F-actin dynamics and apicoplast segregation, and identified two genes critical for host cell egress (Li et al., 2022). Biotin-based proximity-labeling has also been extensively used in *T. gondii* research (Bradley et al., 2020; Kimmel et al., 2021). Examples include in investigation of components of the inner membrane complex (Chen et al., 2015a), calcium-dependent protein kinases and their function during parasite egress (Gaji et al., 2015), the apical annuli- a structure in the parasite cytoskeleton (Engelberg et al., 2020), and components of the tumour-suppressing Hippo pathway, mediating processes such as cytokinesis (Delgado et al., 2021). Bioluminescent *T. gondii* reporter strains have been equally useful for high-throughput drug screening (Key et al., 2020). To our knowledge, bioluminescent technology has not been widely integrated into ‘omics’ studies as a form of screening.

#### Cryptosporidium

*Cryptosporidium* research has incorporated high-content imaging into high-throughput phenotypic screens in the context of drug discovery (Sharling et al., 2010; Love and McNamara, 2020; Love and McNamara, 2021). Luciferase-expressing *Cryptosporidium* parasites (Vinayak et al., 2015; Hennessey et al., 2018) have been used in high throughput screens for parasite inhibitors; and for high-throughput developmental monitoring and genetic tractability (Wilke et al., 2019).

### Kinetoplastids

#### *Trypanosoma* spp.

In the context of Chagas disease, high-content imaging has been used to study host-cell infection rates and drug screening (Alonso-Padilla and Rodríguez, 2014; Alonso-Padilla et al., 2015; Sykes and Avery, 2015; Franco et al., 2019; Fesser et al., 2020; Portella et al., 2021; Svensen et al., 2021). For *T. brucei*, high-content imaging has been used for the measurement of transcriptional activity (Hiraiwa et al., 2018). Proximity-dependent biotinylation approaches have been extremely useful for studying the protein interactome of *T. brucei* in various contexts. These include the identification of bilobe components (Morriswood et al., 2013); changes resulting from the ectopic expression of developmentally-regulated RNA-binding proteins (De Pablos et al., 2017); mapping the interactome of the *T. brucei* cytokinetic machinery (Hilton et al., 2018); identification of key proteins required for microtubule quartet anchorage to basal bodies (Dong et al., 2020); novel cytoskeleton-associated proteins essential for morphogenesis and cytokinesis (Schock et al., 2021); and flagellum tip-specific proteins (Vélez-Ramírez et al., 2021). Still within the umbrella of fluorescence, TrypTag has been an invaluable tool for the parasitology community. Large-scale endogenous tagging of *T. brucei* proteins was performed, and thousands of images generated and made publicly available (Dean et al., 2017). TrypTag aims to document the localization of every protein encoded in the *T. brucei* genome, generated with the aim of validating proteomics analyses, and is an example of the importance and potential of ‘imaging in systems biology’. Bioluminescence use in Kinetoplastid research has been extensive, both as an *in vivo* tool for studying parasite tropism and virulence, and as an *in vitro* high-throughput screening tool. Bioluminescent reporter lines of *T. cruzi* and multiple African trypanosome species have been used to monitor parasite viability in the context of drug and vaccine screening (Hutchens and Luker, 2007; Lewis and Kelly, 2016; Ritchie et al., 2020), but to our knowledge, not within the context of ‘omics’ screens.

#### *Leishmania* spp.

For *Leishmania*, high-content imaging has been used to study anti-leishmanial compounds and their effects on parasites and hosts, comparative anti-parasitic activity against cutaneous and visceral leishmaniasis, or parasite development and infection capacity (Lakshmi et al., 2014; Dagley et al., 2015; Tegazzini et al., 2016; Lamotte et al., 2019; Alcântara et al., 2020; Rosazza et al., 2020; Tirado et al., 2020; Fehling et al., 2021). A recent example of successful integration of imaging in the context of systems biology, was the use of CRISPR-Cas9 genome editing to generate *Leishmania* mutants with altered flagellar function and motility. Dark field microscopy was used to track *Leishmania* swimming, and measure directionality and speed. Bar-seq technology was then used to test fitness mutants within the sandfly vector (Beneke et al., 2019). Another example is the integration of large-scale imaging to investigate morphometric parameters of *Leishmania* during its life cycle, or other cellular landmarks as a reference/basis for post-genomic analyses targeting the parasite’s cell biology (Wheeler et al., 2012; Halliday et al., 2019). These studies set precedence to the current value and future potential of the integration of imaging for high-throughput phenotypic screens. Bioluminescent reporter lines of *Leishmania* spp. have been used to monitor parasite viability in the context of drug and vaccine screening (Caridha et al., 2017; Álvarez-Velilla et al., 2019; Mendes Costa et al., 2019; Agostino et al., 2020; Cohen and Azas, 2021), but to our knowledge, not within the context of ‘omics’ screens.

### Bottlenecks for High-Throughput in Optical and Electron Microscopy and Open Avenues for Future Implementations

The parasitology field has incorporated into its toolbox multiple ‘vanguard’ imaging technologies in optical, force, bioluminescence, and electron microscopy [reviewed in (De Niz et al., 2017)] (**Figure 10**). This includes improved resolution, imaging speed, and hybrid platforms that combine the strengths of more than one technology. So, why have many of these novel imaging methods not been used as high-throughput tools? Three main factors currently prevent several microscopy techniques from becoming high-throughput, and these factors are interrelated. High-throughput imaging relies on fast acquisition, relatively simple outputs, and/or high-throughput data processing. Fast acquisition comes at the expense of spatial and temporal resolution. Due to length restrictions, we focus the discussion below on optical and electron microscopy.

Fluorescence nanoscopy, or super-resolution microscopy, has been revolutionary for cell biology-related fields, including parasitology, and continues to extend its reach into structural biology (de Souza, 2018). Stimulated emission depletion (STED) (Willig et al., 2006), stochastic optical reconstruction microscopy (STORM) (Rust et al., 2006), and structured illumination microscopy (SIM) (Vangindertael et al., 2018) have all been used in the context of Kinetoplastid and Apicomplexan research. Expansion microscopy (ExM) (Chen et al., 2015b) and ultrastructure expansion microscopy (U-ExM) (Gambarotto et al., 2021) have also been incorporated with great success (Amodeo et al., 2021; Bertiaux et al., 2021; Dos Santos Pacheco and Soldati-Favre, 2021; Gorilak et al., 2021; Kalichava and Ochsenreiter, 2021; Liffner and Absalon, 2021; Tomasina et al., 2021). Despite their value, important bottlenecks for using super-resolution in a high-throughput manner are the time required for image acquisition, and the volume of data produced for quantitation. Several adaptations have been designed to address these bottlenecks at the level of microscopy design and image acquisition and processing (**Figure 10**). The former include parallelized STED microscopy, (Bingen et al., 2011; Chmyrov et al., 2013); image scanning microscopy (Schulz et al., 2013); multifocal flat illumination for field-independent imaging (mfFIFI) (Mahecic et al., 2020); and analogue image processing, which increases data acquisition rates up to 100-fold over conventional SIM rates (York et al., 2013). Moreover, novel technologies also continue to advance in optics, imaging, and visualization, which hold important potential for parasitology, and can be incorporated into systems biology approaches at various scales. An example of this is vLUME, which uses virtual reality to enable visualization of single molecule localization (Spark et al., 2020). In addition to advances of the techniques themselves, tools from other fields such as cell biology and bioengineering, have successfully incorporated imaging to their workflow. Microfluidics, organs-on-chip, organoids, and/or tissue bioengineering combined with imaging (**Figure 10**), are important additions to the parasitology toolkit (Mellin and Boddey, 2020; Bernabeu et al., 2021; Sutrave and Richter, 2021). Coupled with advances in image analysis, organoids and spheroids could certainly become valuable tools for ‘omics’ high-throughput validation, and ‘systems biology’ in parasitology (**Figure 10**).

Electron microscopy has also been at the centre of parasitology for decades. Progress in electron microscopy methods over the last couple of decades has been invaluable for cell biology, introducing standalone and hybrid techniques such as focused ion beam scanning electron microscopy (FIB-SEM) (Heymann et al., 2006), scanning transmission electron microscopy (STEM) (von Ardenne, 1938; Kisielowski et al., 2008), cryogenic electron microscopy (cryo-TEM and cryo-EM) (Nakane et al., 2020; Yip et al., 2020), and correlative light and electron microscopy (CLEM) (de Boer et al., 2015). Electron microscopy is extremely labour intensive in terms of sample preparation, image acquisition, and image processing. While still unavailable in parasitology, several platforms have been generated to increase the throughput of electron microscopy (Eberle and Zeidler, 2018; Kornfeld and Denk, 2018; Bykov et al., 2019; Yin et al., 2020). Additional to high-throughput, some of these technologies are considered revolutionary for biology. Cryo-EM is an example, whereby several technical breakthroughs in both, software and hardware, have turned this tool into key for structural biology (Callaway, 2020). The value of this tool has been explored in the context of the *Plasmodium falciparum* proteome, already demonstrating its promising potential within the parasitology field (Beck and Ho, 2021; Chi-Min et al., 2021; Anton et al., 2022).

### Bottleneck for High-Throughput: Progress and Development of Automated Image Analysis

It is important to consider the vast volume of data that the aforementioned methods generate and the importance of image analysis automation. Automated image analysis has been steadily incorporated into cell biology [reviewed in (von Chamier et al., 2019)] and clinical diagnosis in field settings. Beyond diagnosis, the field of parasitology has successfully generated multiple automated image analysis tools. This includes tools developed specifically for Apicomplexan (Kudella et al., 2016; Perez-Guaita et al., 2016; Touquet et al., 2018; Fisch et al., 2019; Bauman et al., 2020; Fisch et al., 2020; Hung et al., 2020; Dey et al., 2021; Shaw et al., 2021; Yoon et al., 2021) and Kinetoplastid research (Wheeler et al., 2012; Moon et al., 2014; Yazdanparast et al., 2014; Gomes-Alves et al., 2018; Moraes et al., 2019; Wheeler, 2020) and applied to a range of questions, from micrograph analysis, subcellular landmark investigation and parasite motility, to insect vector behavior. Many of these parameters are common outputs from ‘omics’ and large screen studies. HRMAn (Host Response to Microbe Analysis) is a high-throughput, high-content, single-cell image analysis platform which incorporates machine learning and deep convolutional neural networks (Fisch et al., 2019). Classification of features based on datasets has been used to distinguish phenotypic patterns of host-protein recruitment; detection and quantification of *T. gondii-*containing vacuoles; and analysis of host cell responses to *T. gondii* infection. More recent updates (HRMAn 2.0) offer the possibility to investigate 3D information, and applications to other pathogens beyond *T. gondii* (Fisch et al., 2021).

Despite the increased availability of image analysis tools, two ‘hurdles’ stand in the way of their widespread use among the parasitology community. The first is tool availability. Open source software has been pivotal for research, with ImageJ (Schneider et al., 2012), CellProfiler (Carpenter et al., 2006), Ilastik (Berg et al., 2019), OMERO (Allan et al., 2012; Li et al., 2016), ICY (Chaumont et al., 2011) being some examples of the most used tools for image analysis. A step, which remains to be more widely implemented in the parasitology imaging field is the existence of open repositories for code and image databases of both parasites and hosts, equivalent to VEuPathDB (Amos et al., 2022) resources, and under the lines of TrypTag (Dean et al., 2017). The second hurdle is tool accessibility: if the tools are available, can everyone in the parasitology community use them? We envisage that a step to achieve this is the development of user-friendly environments that allow users with little or no coding experience to use these resources. Recent successful examples include LOBSTER (Tosi et al., 2020), BIAFLOWS (Rubens et al., 2020), OpSeF (Rasse et al., 2020), and ZeroCostDL4Mic (von Chamier et al., 2021). Moreover, specialists are needed to bridge the two disciplines (artificial intelligence in imaging, and biology). A successful example in biology of such a training initiative is NEUBIAS (Martins et al., 2021).

Altogether, we envisage that advances in microscopy will allow its extensive integration into ‘omics’-based studies and functional screens, as a valuable tool to further our knowledge on parasite biology.

## Further Insights

### Big Data in Context: How Big Is ‘Big Data’ in Parasitology, and What Can We Learn From Other Fields?

While we have addressed ‘big data’ in parasitology, it is worth asking how does such data compare to other research fields; what can we learn from other fields; and how will the growing scale of the data in parasitology be stored and handled computationally. Amongst the most striking example of big data generation is the European Council for Nuclear Research (CERN), with one of the most highly demanding computing environments in research, handling over 330 petabytes – and envisaging over the next decade, to require data storage capacities in the order of exabytes (10^18^ bytes). Its computing demands fostered the creation of vital resources such as the world wide web, which was initiatlly conceived to facilitate data sharing amongst scientists worldwide. At the heart of CERN’s infrastructure are now the worldwide large hadron collider (LHC) computing grid, which gives physicists worldwide near-real-time access to LHC data (Geddes, 2012; Britton and Lloyd, 2014); the CERN Openlab, a public-private partnership through which CERN collaborates with information and communication technology (ICT) leading companies (such as Oracle, Micron, Intel, Siemens, Google, and IBM, among others); the data preservation in high energy physics (DPHEP) collaboration; and the CERN Internet eXchange Point (CIXP) among others. Other examples of large-scale research-focused collaborations include the 1,000 and 100,000 genomes projects. The 1000 genomes project aims at cataloguing common human genetic variation (Abecasis et al., 2010). For this project, several tools were developed and deployed to allow for widespread data access. This included the creation of a Data Coordination Centre (DCC), set up by the European Bioinformatics Institute (EBI) and the National Centre for Biotechnology (NCBI) to manage the data, and facilitate community access (Clarke et al., 2012). The 100,000 genomes project is an initiative to sequence genomes from thousands of patients affected by rare diseases, or cancer, and provide insights into the role of genomics in health/disease, and pave the way toward personalized medicine (Turnbull et al., 2018). What these major projects with ‘big’ data have in common is that they catalysed the generation of extraordinary tools at the level of hardware and software to ensure real-time open access by the scientific community as well as relevant data storage, maintenance and preservation capacities. Equally, these projects are based on international private and public partnerships from various sectors (including healthcare, governmental organizations, and information technology/computing developers). While generating such tools for parasitology alone might be a complex task, with these examples we aim to highlight the avenues that remain to be explored as well as the tools that already exist and could be capitalized on for our field and our understanding of both parasite and host.

### VEuPathDB and Its Role in Parasitology

A revolutionary resource in the parasitology field is VEuPathDB. This integrated database provides a repository of datasets across all “-omics” disciplines that can be accessed for free (Warrenfeltz et al., 2018), ensuring equitable access to these resources. VEuPathDB is, however, not only a database, as the web-based service includes tools available for data searching, visualisation and comparisons. Moreover, the founding team is committed to democratizing science and frequently offer training world-wide to allowed hundreds of researchers across the global North and South, to access the full capacity of this tool. The online tutorials available through the resource also ensure that users with limited bioinformatics experience are able to mine the data deposited. Altogether, VEuPathDB has been committed to promoting equitable access to data generated across our field.

## Concluding Remarks

**Figure 11:**
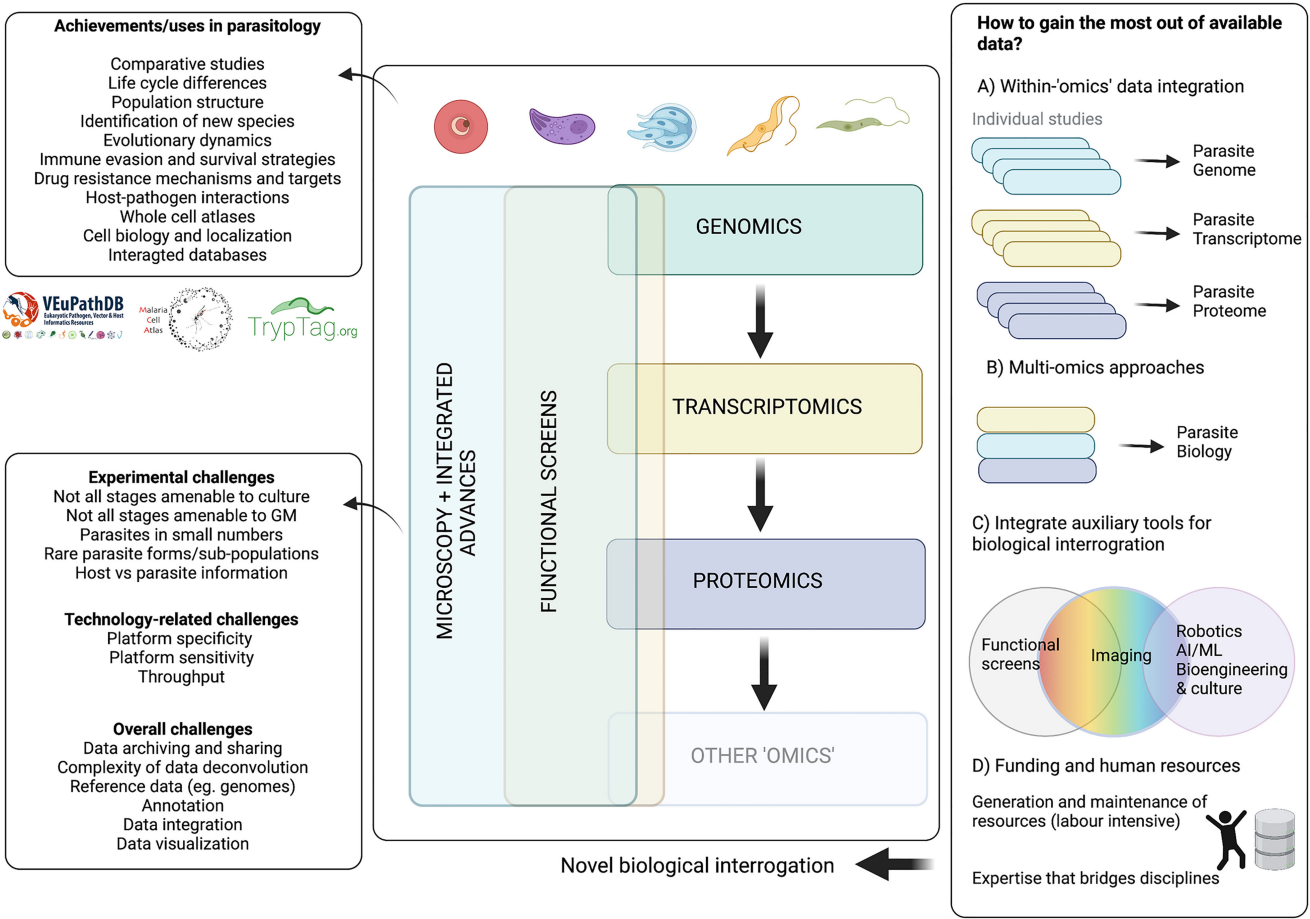
**‘**Omics’ technologies in parasitology: challenges and future directions. Genomics, transcriptomics and proteomics (as well as other ‘omics’ technologies), have allowed us to study a plethora of questions in parasitology. While bulk-based ‘omics’ have contributed greatly to parasitology, single cell-based ‘omics’ approaches have highlighted unexpected heterogeneity in parasite populations. Given the complex life cycles of Apicomplexan and Kinetoplastid parasite species, understanding the interconnection between parasite and host is key. ‘Omics’ technologies that allow the simultaneous investigation of parasite and host will continue to play important roles in understanding host-pathogen interactions, including topics of current major interest such as tissue tropism; immune evasion; parasite latency, dormancy, and persistence; and parasite-host circadian rhythms, among others. Functional screens based on genome mutagenesis, RNA regulation and protein regulation, have become vital tools for investigating parasite biology. Together with auxiliary technologies, such as high-throughput imaging, functional screens are powerful tools for parasitology. Current advances in microscopy are allowing valuable low-throughput techniques (eg. super-resolution, and electron microscopy), to be adapted for higher throughput. Together with the incorporation of robotics and artificial intelligence, these valuable tools could become suitable for integration into ‘omics’ research and functional screens. Several challenges remain for ‘omics’ in parasitology, including experimental, technological, and overall challenges. Among the latter are the need for better data annotation and integration. We envisage that vital for an improved/increased use of available and future ‘omics’ data are within-omics data integration, multi-omics data integration, and multi-disciplinary data integration. Equally funding and dedicated personnel to the creation, maintenance, annotation, curation and update of available data is vital. A successful transition in this respect will enable improved collaborative science, and addressing novel and outstanding biological questions. Figure created with BioRender.com.

In this review, we have explored ‘omics’-based technologies and their major contributions to parasite biology (summarized in **Figure 11**). Addressing the fundamental question: how can we gain the most from the abundance of currently existing and future ‘big data’? As ‘omics’ technologies progress and more studies incorporate them in their research questions, a risk that exists is the accumulation of data whose biological wealth is not fully explored or exploited. To avoid this, we require an advancement in data management/integration, and potentially, a change in research practices towards ‘big data’. Improved annotation of reference genomes is key for ‘omics’ technologies, as is the need for constantly updated reference resources. This is a human resource-intensive task that should not be underestimated in its requirements for dedicated personnel and funding. Moreover, a research philosophy that envisages better data integration practices, at single ‘omics’ and ‘multi-omics’ levels, is vital. Improved accessibility is key for such integration. This comes in the form of improved data availability, improved data visualization tools, and a focus on training next generations of scientists to include expertise that bridges disciplines (eg. bioinformatics, artificial intelligence and biology). Together, ‘omics’ approaches and functional screens have pushed the boundaries of our knowledge on parasite biology, and we envisage that the aforementioned investments are some of many that will allow us to take the most advantage of current and future ‘big data’.

## Author Contributions

All authors listed have made a substantial, direct, and intellectual contribution to the work, and approved it for publication.

## Conflict of Interest

The authors declare that the research was conducted in the absence of any commercial or financial relationships that could be construed as a potential conflict of interest.

## Publisher’s Note

All claims expressed in this article are solely those of the authors and do not necessarily represent those of their affiliated organizations, or those of the publisher, the editors and the reviewers. Any product that may be evaluated in this article, or claim that may be made by its manufacturer, is not guaranteed or endorsed by the publisher.
